# Hydrolyzed Fat Formula Increases Brain White Matter in Small for Gestational Age and Appropriate for Gestational Age Neonatal Piglets

**DOI:** 10.3389/fped.2020.00032

**Published:** 2020-02-12

**Authors:** Megan P. Caputo, Jennifer N. Williams, Jenny Drnevich, Emily C. Radlowski, Ryan J. Larsen, Bradley P. Sutton, Brian J. Leyshon, Jamal Hussain, Manabu T. Nakamura, Matthew J. Kuchan, Tapas Das, Rodney W. Johnson

**Affiliations:** ^1^Division of Nutritional Sciences, University of Illinois, Urbana, IL, United States; ^2^Department of Animal Sciences, University of Illinois, Urbana, IL, United States; ^3^High Performance Biological Computing Group and the Carver Biotechnology Center, University of Illinois, Urbana, IL, United States; ^4^Beckman Institute, University of Illinois, Urbana, IL, United States; ^5^Department of Bioengineering, University of Illinois, Urbana, IL, United States; ^6^Department of Food Science and Human Nutrition, University of Illinois, Urbana, IL, United States; ^7^Abbott Nutrition, Discovery Research, Columbus, OH, United States; ^8^Neuroscience Program, University of Illinois, Urbana, IL, United States

**Keywords:** intrauterine, growth, brain, piglets, myelin, lipids

## Abstract

**Background:** Intrauterine growth restriction is a common cause of small for gestational age (SGA) infants worldwide. SGA infants are deficient in digestive enzymes required for fat digestion and absorption compared to appropriate for gestational age (AGA) infants, putting them at risk for impaired neurocognitive development.

**Objective:** The objective was to determine if a hydrolyzed fat (HF) infant formula containing soy free fatty acids, 2-monoacylglycerolpalmitate, cholesterol, and soy lecithin could increase brain tissue incorporation of essential fatty acids or white matter to enhance brain development in SGA and AGA neonatal piglet models.

**Methods:** Sex-matched, littermate pairs of SGA (0.5–0.9 kg) and AGA (1.2–1.8 kg) 2 days old piglets (*N* = 60) were randomly assigned to control (CON) or HF formula diets in a 2 × 2 factorial design. On day 14, 24 piglets were used for hippocampal RNA-sequencing; the rest began a spatial learning task. On days 26–29, brain structure was assessed by magnetic resonance imaging (MRI). Cerebellum and hippocampus were analyzed for fatty acid content.

**Results:** SGA piglets grew more slowly than AGA piglets, with no effect of diet on daily weight gain or weight at MRI. HF diet did not affect brain weight. HF diet increased relative volumes of 7 brain regions and white matter (WM) volume in both SGA and AGA piglets. However, HF did not ameliorate SGA total WM integrity deficits. RNA sequencing revealed SGA piglets had increased gene expression of synapse and cell signaling pathways and decreased expression of ribosome pathways in the hippocampus compared to AGA. HF decreased expression of immune response related genes in the hippocampus of AGA and SGA piglets, but did not correct gene expression patterns in SGA piglets. Piglets learned the T-maze task at the same rate, but SGA HF, SGA CON, and AGA HF piglets had more accurate performance than AGA CON piglets on reversal day 2. HF increased arachidonic acid (ARA) percentage in the cerebellum and total ARA in the hippocampus.

**Conclusions:** HF enhanced brain development in the neonatal piglet measured by brain volume and WM volume in specific brain regions; however, more studies are needed to assess long-term outcomes.

## Introduction

Neonatal mortality accounts for 45% of deaths in children under 5 years old worldwide (1), and ~22% of neonatal deaths in low and middle income countries are small for gestational age (SGA) infants (2). The cause of SGA birth is often intrauterine growth restriction (IUGR), which leads to complications such as neonatal infections, hypoglycemia, difficulty feeding, and hypothermia (2). In addition to these immediate problems, SGA infants have an increased risk of delayed neurodevelopment (3). Many studies have examined the cognitive development and behavior of infants born SGA. Pryor et al. (4) found that adolescents born SGA were more likely to have poor concentration, antisocial behavior, and lower mean IQ scores than their appropriate for gestational age (AGA) counterparts. Another study reported that adults who were born SGA had mild but significant deficits in academic, professional, and economic achievement (5). A comprehensive prospective study by Geva et al. (6) concluded that most children with IUGR had difficulty with learning and memory, which was more pronounced when catch up growth did not occur. A neonatal piglet model of SGA due to IUGR developed by Radlowski et al. (7) demonstrated deficits in learning and memory along with white matter (WM) deficits that persisted even with catch up growth.

Brain expansion and white matter deposition involve accretion of large quantities of fat, a process that occurs at a time when infants naturally have lower capability for fat digestion and absorption. An infant formula that contains hydrolyzed fat may increase dietary fat absorption and enhance white matter deposition in both the SGA and AGA infant brain. Breast milk and formulas both contain triacylglycerols, which consist of three fatty acids bound to glycerol, as a major source of energy (8). Traditional formulas provide fat in triacylglycerol form from vegetable oils. Breast milk contains bile salt-stimulated lipase, which contributes to the hydrolysis of triacylglycerols into fatty acids and monoacylglycerols in the stomach and small intestine to facilitate absorption (9, 10). Thus, hydrolyzed fat formula circumvents the technical challenges of lipase addition to formula by providing free fatty acids and monoacylglycerols, the constituents of triacylglycerols. These components may improve lipid absorption in SGA and AGA infants (11). Adequate lipid absorption and tissue accretion during early post-natal life is critical, as the long chain polyunsaturated fatty acids (LC-PUFA) arachidonic acid (ARA) and docosahexaenoic acid (DHA) play key roles in proper neurocognitive development [reviewed in (12)]. While exclusive breast feeding is recommended by the World Health Organization (WHO) for infants up to 6 months of age, only 38% of infants worldwide have caregivers that follow these guidelines (13). Reasons for low breastfeeding rates include difficulty lactating and concerns about the infant's weight and nutritional status (14). The current study applied a hydrolyzed fat system designed to enhance fatty acid absorption and deposition in the brain of neonatal piglets born AGA and SGA. Our objective was to ameliorate previously demonstrated brain structural and behavioral deficits in SGA piglets.

## Materials and Methods

### Animals

**Figure 1:**
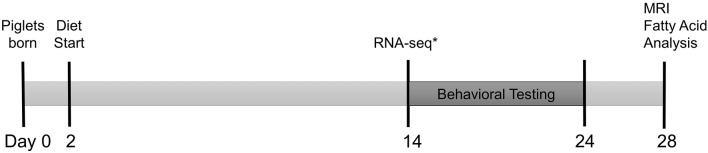
Experimental timeline. Full term, naturally delivered, sex-matched littermate pairs of piglets (*n* = 30 SGA, *n* = 30 AGA) were obtained from the University of Illinois Swine Farm at 2 days old and placed onto either CON or HF diet. Piglets began T-maze behavioral testing at 14–17 days old, tested for 9 days, and underwent magnetic resonance imaging (MRI) and tissue collection for hippocampal and cerebellar fatty acid analysis at 26–29 days old. *A separate cohort of identically raised piglets (*n* = 6/group) were sacrificed at 14 days old for hippocampal RNA-sequencing. AGA, appropriate for gestational age; SGA, small for gestational age; CON, control; HF, hydrolyzed fat.

Piglets were PIC Camborough (dam) × PIC 359 (sire), full term, naturally delivered, sex-matched littermate pairs (*n* = 30 SGA, *n* = 30 AGA) obtained from the University of Illinois Swine Farm at 2 days old to allow colostrum consumption. Piglets underwent minimal routine processing on the farm by remaining intact, but received an iron dextran (cat. No. 014159, Henry Schein Animal Health, Dublin, OH, USA) and antibiotic injection (Gentamicin Piglet Injection, Agri Laboratories, Ltd., St. Joseph, MO, USA) per routine farm practice and as directed on the labels. SGA was defined as piglets weighing 0.5–0.9 kg at birth, and AGA was defined as piglets weighing 1.2–1.8 kg at birth. These weight ranges were established previously by compiling birth records from the Imported Swine Research Laboratory over 3 years (15). Piglets were placed individually into a caging system under standard conditions as described in a previous publication (16), and randomly assigned to HF or CON diet treatment groups in a 2 × 2 factorial arrangement of size (SGA or AGA) and diet (CON or HF). These piglets were used for all analyses described below with the exception of RNA-sequencing, for which a separate but equally treated replicate of piglets was used. Figure 1 summarizes the experimental timeline. All animal care and experimental procedures were in accordance with the National Research Council Guide for the Care and Use of Laboratory Animals and approved by the University of Illinois at Urbana-Champaign Institutional Animal Care and Use Committee.

### Diet

Diets were formulated to meet the nutritional needs of neonatal piglets and were supplied in a premixed, ready to feed format (Table 1) by Abbott Nutrition (Columbus, OH, USA). CON formula contained 100% triglyceride rich oil (39% high oleic safflower oil (HOSO), 29% soy oil, and 28% coconut oil). HF formula contained 50% triglyceride (35% HOSO, 15% coconut oil), 18% soy FFAs, 20% monoacylglycerol palmitate, and 10% soy lecithin. To acclimate the piglets to the fat content, three study formulas were used to create the experimental diets and the piglets were fed a 70%/30% blend of full fat (CON or HF)/very low fat diet for the first 2 days, followed by an 80%/20% blend on days 3–4, and a 90%/10% blend on day 5 before being fed 100% full fat on day 6 until the end of the study. Piglets were weighed each morning and provided the liquid diet (300 mL formula/kg body weight/d) in 5 equal bolus feedings given at 09:00, 13:00, 16:00, 19:00, and 22:00. Meals were provided in bowls mounted in each piglet's cage to acclimatize the piglets to eating from a bowl, which is essential for performing the T-maze task. Diets were stored at 4°C and any excess was disposed of each night. Supplemental water was not provided aside from that in the diet.

**Table 1:** Nutrient composition of study formulas (Per L as Fed).

	**CON**	**HF**	**Very low fat**
**Energy content, cal/g**	1.09	1.09	0.76
**Protein content, %**	5.5	5.5	5.5
**Fat content, %**	7.02	7.02	0.34
**Protein, g**	56.5	57.1	58.1
**Fat, g**	71	71	3.7
High Oleic Safflower Oil, g	27.66	24.75	0
Soy oil, g	20.7	0	0
Coconut oil, g	19.8	10.5	0
Mono-acyl glycerol palmitate, g	0	14.25	0
Soybean oil free fatty acids, g	0	12.4	0
Lecithin, g	0	7.3	0
Distilled monoglycerides, g	1.5	0	0
DHA, mg	146	135	161
ARA, mg	312	322	355
Cholesterol, mg	0	360	0
Carotenoids, mg	59	59	61
Remaining fat from proteins, carrier oils from vitamins, carotenoids, DHA, and ARA, g	0.82	1.28	3.12
**Carbohydrate, g**	62	63	138
**Vitamins**			
Vitamin A Palm, IU	6,146	6,677	5,584
Vitamin E, mg	18	18	19
Vitamin C, mg	375	375	375
B1, mg	2.45	2.25	2.45
B2, mg	6.1	6.02	6.7
B6, mcg	783	802	832
B12, mcg	10.2	10.1	11.4
Pantothenic acid, mg	12	12.38	12.7
Folic acid, mcg	334	297.4	328.2
Niacin, mg	16.4	16.22	17
Biotin, mcg	118	115.4	125.9
Total Choline, mg	317	474	341
**Minerals**			
Sodium, mg	762	755	802
Potassium, mg	2,228	2,319	2,539
Chloride, mg	1,175	1,175	1,184
Calcium, mg	2,402	2,371	2,432
Phosphorus, mg	1,269	1,362	1,280
Magnesium, mg	154	158.1	158.2
Iron, mg	20	20.2	21.2
Zinc, mg	13.1	12.69	13.77
Copper, mg	1.13	1.09	1.18

### Magnetic Resonance Imaging (MRI)

Piglets (AGA CON *n* = 8, AGA HF *n* = 8, SGA CON *n* = 9, SGA CON *n* = 9) were scanned at 26–29 days old to estimate brain region volumes, WM and gray matter (GM) composition, WM integrity, and metabolite concentrations. All scanning was conducted at the Biomedical Imaging Center at the Beckman Institute (University of Illinois, Urbana, IL, USA) using a Siemens MAGNETOM Trio 3T imager with a Siemens 32-channel head coil (Siemens, Erlangen, Germany). Upon reaching the MRI facility, pigs were anesthetized using a telazol:ketamine:xylazine (TKX) solution (100/50/50 mg/kg; Fort Dodge Animal Health, Overland Park, KS, USA). TKX was administered intramuscularly at 0.022 mL/kg body weight, and anesthesia maintained using isoflurane (98% oxygen/2% isoflurane) administered via nose mask. An MRI compatible pulse oximeter was used to monitor piglet vital signs every 5 min. Once fully anesthetized, piglets were placed in dorsal recumbency and wrapped with warmed blankets. Once neuroimaging procedures were complete, pigs were transported back to the animal facility for euthanasia. Pigs were sedated using TKX (0.022 mL/kg body weight, i.m.) followed by intracardiac injection of sodium pentobarbital (72 mg/kg body weight Fatal Plus, Vortech Pharmaceuticals, Dearborn, MI, USA). Brains were extracted after euthanasia by removing the head, creating a skin incision to expose the sagittal suture of the skull and retracting the skin bilaterally to expose the frontal and parietal bones. A bone saw was used to create a window rostrally at the level of the medial canthus of the eyes, caudally at the level of the ear pinnae, and laterally above each orbit. The brain was then removed by severing the cranial nerves at their foramen with a stainless steel weighing spatula and gently sliding the brain into a pre-weighed weigh boat. The right hippocampus and cerebellum were then dissected out and flash frozen on dry ice and stored at −80°C until fatty acid extraction and analysis could be performed.

For structural analyses of brain growth (i.e., volume of discrete brain regions), anatomic images were acquired using a 3D T1-weighted magnetization-prepared rapid gradient-echo sequence with the following parameters: repetition time = 1,900 ms; echo time = 2.48 ms; inversion time = 900 ms, flip angle = 9°, matrix = 256 × 256, slice thickness = 0.7 mm. The final voxel size was 0.7 mm isotropic across the entire head from the tip of the snout to the cervical/thoracic spinal cord junction as described previously (17).

Diffusion tensor imaging (DTI) was performed with the following parameters: repetition time = 5,000 ms, echo time = 91 ms, matrix = 100 × 100, field of view = 200 mm × 200 mm, 40 slices of 2 mm thickness, 31 diffusion directions with *b* = 1,000 s/mm^2^, 3 averages.

Single-voxel spectroscopy (SVS) was performed using a Point RESolved Spectroscopy (PRESS) sequence with the following parameters: repetition time = 3,000 ms, echo time = 30 ms, flip angle = 90°, bandwidth = 2,000 Hz, 1,024 points, water suppression (50 Hz), frequency shift = −2.0 ppm, voxel size = 12 mm × 12 mm × 25 mm, 128 averages, and six regional saturation bands adjacent to each face of the voxel. Data for water-scaling was obtained in a second scan with the same imaging parameters, but no water-suppression, 8 averages, and frequency shift = 0 ppm. The voxel was placed over the hippocampi, as shown in Supplementary Figure S1. A representative spectra is shown in Supplementary Figure S2.

Software and methods used for image processing for voxel-based morphometry (VBM) and brain region volume estimation analysis were utilized as previously described (7), with the exception of the use of Statistical Parametric Methods (SPM8; Wellcome Department of Imaging Neuroscience, Institute of Neurology, London, UK) for VBM analysis (described below). One SGA CON file was removed from analysis due to abnormal appearance of the cerebellum. DTI was analyzed using the diffusion toolbox in the FMRIB Software Library (FSL) software package as previously described (18) to obtain values of fractional anisotropy, axial diffusivity, radial diffusivity, and mean diffusivity of cortical white matter, caudate, corpus callosum, cerebellum, internal capsule, thalamus, and both hippocampi.

### Fatty Acid Analysis

Lipid extraction and analysis of fatty acid species were performed as described previously (19). Briefly, ~100 mg of piglet cerebellum and hippocampus were weighed. Then, lipids were extracted from the samples, and fatty acids were trans-methylated in a single step. Fifty μg of 1,2-diheptadecanoyl-sn-glycero-3-phosphocholine (Avanti Polar Lipids, Alabaster, AL) were added to each sample as an internal standard. Fatty acid methyl esters were separated and quantitated using a GC-2010 Plus gas chromatograph (Shimadzu, Columbia, MD) equipped with a DB-FFAP capillary column (Agilent Technologies, Santa Clara, CA). Fatty acid data are presented as percent weight of total fatty acids and mg/g tissue.

### Hippocampal RNA Extraction and Sequencing

An independent cohort of neonatal piglets (*n* = 6/group) was used to analyze the effects of SGA and HF on the hippocampal transcriptome. Piglets were raised and treated identically to those used for cognitive testing and MRI, but at 14 days old, piglets were euthanized and hippocampi were dissected and stabilized with RNAlater (Qiagen, Germantown, MD, USA), snap frozen, and stored at −80°C until RNA could be extracted. Tissue (~50 mg) was homogenized in 1 mL TRIzol Reagent (ThermoFisher Scientific, Waltham, MA, USA) following manufacturer's protocol steps 1–8. After step 8, 0.4 mL of 200 proof ethanol was added and the samples briefly vortexed. The samples were loaded into RNeasy Mini Kit columns (cat. No. 74104, Qiagen, Germantown, MD, USA) and the manufacturer's protocol followed from Part 1 step 3. Genomic DNA was removed with the RNase-free DNase Set (cat. No. 79254, Qiagen, Germantown, MD, USA) following manufacturer's protocol. Samples were analyzed in the DNA Services laboratory of the Roy J. Carver Biotechnology Center (University of Illinois, Urbana, IL, USA) using AATI Fragment Analyzer (Advanced Analytics, Ames, IA, USA) to determine RNA integrity and the presence/absence of genomic DNA. Genomic DNA was not detected, and the average RNA quality number (RQN) of the samples was 5.3 due to the presence of pre-spliced RNA. RNAseq libraries were then constructed and sequenced using the TruSeq LT Stranded RNA Sample Preparation Kit (Illumina, Inc., San Diego, CA, USA). PolyA+RNA was selected from 1,000 ng total RNA provided, then first strand synthesis was synthesized with a random hexamer and SuperScript II (Life Technologies, Grand Island, NY, USA). Double stranded DNA was blunt-ended, 3'-end A-tailed and ligated to indexed adaptors. The adaptor-ligated double-stranded cDNA was amplified by PCR for 10 cycles with the Kapa HiFi polymerase (Kapa Biosystems, Woburn, MA). Final libraries were quantitated by using Qubit High-Sensitivity DNA (Life Technologies, Grand Island, NY, USA) and average size determined on the AATI Fragment Analyzer. Libraries were pooled evenly and cleaned one additional time using a 50:50 ratio with AxyPrep Mag PCR Cleanup beads (Axygen, Inc. Union City, CA) to ensure removal of primer and adaptor dimers, then evaluated on AATI Fragment Analyzer. The final pools were diluted to 5 nM concentration and further quantitated by qPCR on a BioRad CFX Connect Real-Time System (Bio-Rad Laboratories, Inc. CA, USA). The final pool containing 24 libraries was denatured according to Illumina protocols and loaded onto 1 lane of an 8-lane flowcell at a concentration of 300 pM for cluster formation on the cBOT and then sequenced from one end of the fragments on the HiSeq4000 with version 1 SBS sequencing reagents for a total read length of 100 nt with perfect quality scores (Solexa scale = 40). Fastq files were generated, compressed, and demultiplexed from.bcl files with the bcl2fastq v2.17.1.14 Conversion Software (Illumina, Inc., San Diego, CA, USA).

### Alignment and Statistical Analysis

Alignment and statistical analysis was done by the High-Performance Biological Computing group (HPCBio, University of Illinois, Urbana, IL, USA). Residual adapter content and low quality bases were removed using Trimmomatic (version 0.33) with the following parameters: ILLUMINACLIP:TruSeq3-SE.fa:2:15:10 LEADING:28 TRAILING:28 SLIDINGWINDOW:4:15 MINLEN:30. The trimmed reads were aligned to NCBI's *Sus scrofa* 10.2 reference genome and annotation release 105 using STAR (version 2.5.2a). Approximately 82–86% of reads aligned. All gene counts were generated using featureCounts from the subread (version 1.5.0) package using –s 2 –t exon and –g gene_id parameters, where gene_id were Entrez Gene IDs that were manually pulled from the Dbxref attribute in the gene annotation release 105 gff file. Between 6.0 and 9.2 million reads per sample were assigned to genes.

All analyses hereafter were done in R (75; v 3.3.3). The numbers of reads per gene were normalized using TMM normalization (20) in the edgeR package (21). 20,940 genes without 1 count per million in at least 3 samples were removed from further analysis, leaving 17,942 genes to be analyzed for differential expression. The limma package's (22) “voom” method (23) was used to assess a statistical model that included terms for size, diet, size*diet, the batch effect of sex and five surrogate variables (sv) estimated by surrogate variables analysis (24, 25). These sv correct for nuisance covariance structures found in the data set such as those due to cohort, pair variation within a cohort and individual sample effects not strong enough to be called true outliers. Seven different contrasts were pulled from the model: the main effects of size and diet, the interaction term and the four logical pairwise comparisons. Multiple hypothesis testing adjustment using the False Discovery Rate (FDR) method (26) was done globally across all seven contrasts together, so that the same raw *p*-value ended up with the same FDR *p*-value in all contrasts. Function annotation information for each gene was pulled from two sources. Bioconductor's (27) org.Ss.eg.db package was used to get Gene Ontology's Biological Process, Cellular Component and Molecular Function terms for *Sus scrofa* genes. Updated KEGG pathways, matched based on Entrez Gene IDs, were pulled directly from KEGG using the KEGGREST package.

### Pathway Analysis

For visualization of differentially expressed genes (DEGs), a heat map was created for genes that had a FDR *p* <0.4 in at least one of the seven contrasts. We used a larger FDR threshold than normal to gain a broader view of the gene expression patterns. Each column represents an individual pig, and each row represents the relative expression level (row z-score) of one gene across all pigs. The columns were clustered based on similarity of expression patterns of each individual pig. Functional enrichment analysis was performed using DAVID v6.8 (28). Entrez IDs of up and down-regulated DEG were uploaded and analyzed separately using the entire set of transcribed genes specific to our samples as the background. Default settings of annotation categories and sources for DAVID were accepted. The defaulted stringency settings were used for Gene Ontology (GO) enrichment analysis. Enriched GO terms (cellular component, molecular function, and biological process) and KEGG pathways were reported.

### T-Maze Task

To test the effects of SGA status and diet on learning and memory, a T-maze task designed for piglets was administered as previously described (7, 16, 29, 30). Testing began when piglets (AGA CON *n* = 5, AGA HF *n* = 5, SGA CON *n* = 6, SGA HF *n* = 6) were a minimum of 14 days old. Piglets are a precocious species and learn the maze at a young age, however the growth rate and size of the piglets limited testing to <4 weeks of age. Piglets were acclimated to handling, the chocolate milk reward, and the bowl covers 3 days prior to the start of testing to prevent neophobia. Chocolate powder (Nesquik No Added Sugar, Nestle S.A., Vevey, Vaud, Switzerland) was mixed with the diets (HF or CON) at 20 g/ 300 mL of formula. Trials were recorded using a Sony Handycam (model # DCR-SR300, Sony Corporation, Minato, Tokyo, Japan) and EthoVision XT version 3.1 tracking software (Noldus Information Technology, Inc., Leesburg, VA, USA).

### Statistical Analysis

Data analysis for weight gain and T-maze task were conducted using the MIXED procedure of the SAS 9.4 software (SAS Institute, Cary, NC, USA) as a 3-way (size*diet*day) repeated measures ANOVA for weight gain and 3-way (size*diet*day) ANOVA for the T-maze task. Tukey's *post hoc* test was used to determine which groups were different when interactions were significant. The slice option was used to determine which groups were different when a 3-way interaction was significant.

Statistics for brain fatty acid analysis were performed using a 2-way ANOVA (size*diet) in SPSS16 (SPSS Inc., Chicago, IL, USA).

Analysis of final body weights, final brain weights, and brain to body weight ratio were performed using the MIXED procedure of SAS 9.4 as a 2-way (size*diet) ANOVA with Tukey's *post hoc* test. Outliers were removed if assumptions of normality were not met by removing data points with internally studentized residuals outside the ± 2 range.

Statistical analysis of brain volumes and DTI values were performed using the MIXED procedure of SAS 9.4 as a 2-way (size*diet) ANOVA with Tukey's *post hoc* test. Outliers were removed if assumptions of normality were not met by removing data points with internally studentized residuals outside the ± 2 range.

Analysis of single voxel spectroscopy, normalized by the non-suppressed water signal, was conducted using LCModel (version 7.3-0L) (31). Data were excluded from the analysis if the Cramer-Rao lower bound exceeded 20%. Results were then analyzed using a 2-way (size*diet) ANOVA with Tukey's *post hoc* test in GraphPad Prism v.7. Outliers were removed using the ROUT algorithm in Prism set to Q = 5% if assumptions of normality were not met.

VBM was analyzed using the statistical parametric methods SPM8 program. The analysis was conducted following the procedures outlined in the SPM8 Manual by Ashburner et al. (32) and in the VMB8 Manual by Kurth et al. (33). A full factorial design was selected, with the first factor set to birth weight, with two levels set to AGA and SGA and the second factor set to diet, with two levels set to CON and HF. ANCOVA was selected for each factor (“ANCOVA by effect”), and global normalization was set to ANCOVA to control for nuisance effects. No covariates and no grand mean scaling were selected. Pseudo-F and Pseudo-T statistic maps were generated showing areas where there was a difference in GM or WM using an uncorrected *p* ≤ 0.001. A threshold of at least 20 edge-connected voxels (clusters) was used.

All graphs were created using GraphPad Prism v.7 with the exception of the RNA-sequencing heat map (described above). Data are presented as mean ± SEM. Significance was set at *p* < 0.05.

## Results

### HF Diet Does Not Affect Growth or Brain Weight of Neonatal Piglets

**Figure 2:**
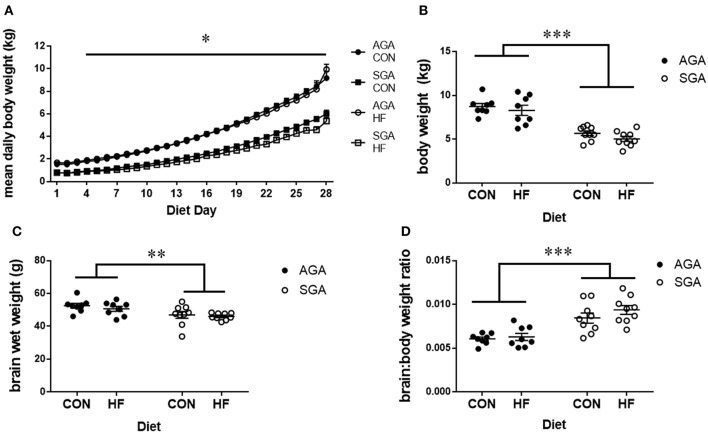
Brain and body weights of piglets fed a control or HF formula. Body weight was measured each morning **(A)**. SGA piglets gained weight in a similar pattern but a slower rate than AGA. AGA piglets began gaining weight at day 4, while SGA piglets did not show appreciable weight gains until day 7. AGA piglets outweighed SGA piglets every day for the remainder of the study starting at day 4. Diet did not affect piglet weight gain. SGA piglets weighed less than AGA on the day of MRI regardless of diet **(B)**. Brain weights taken after the MRI showed that SGA piglets had smaller brains **(C)**. The brain to body weight ratio was greater in SGA piglets. There were no effects of diet on brain weights or brain to body weight ratio **(D)**. AGA, appropriate for gestational age; SGA, small for gestational age; CON, control; HF, hydrolyzed fat. Data are presented as mean ± SEM, AGA *n* = 8 per group, SGA *n* = 9 per group. ***p < 0.001, **p < 0.01, *p < 0.05.

The HF diet was well-tolerated by all piglets. There was a day*size interaction since SGA gained weight in a similar pattern but a slower rate than AGA (*p* < 0.001; Figure 2A). Tukey's *post hoc* test revealed body weight increases in AGA piglets beginning at day 4 compared to day 1 (*p* = 0.046), while SGA piglets did not show appreciable weight gains until day 7 compared to day 1 (*p* = 0.039). SGA and AGA piglet body weight did not differ from each other until day 4 (*p* = 0.041), at which point AGA piglets outweighed SGA piglets every day for the remainder of the study. Diet did not affect piglet weight gain (*p* = 0.351).

Final body weights were taken on the morning of MRI scanning. There were main effects of SGA (*p* < 0.001; Figure 2B), with no effect of diet (*p* = 0.17) on final body weight. Brain weights taken after MRI (Figure 2C) showed a main effect of SGA (*p* = 0.002) but not diet (*p* = 0.36). The brain to body weight ratio (Figure 2D) was greater in SGA piglets (*p* < 0.001). There was no effect of diet (*p* = 0.22) on the brain to body weight ratio.

### HF Diet and SGA Status Affect Structural Volumes, WM, and GM Composition as Measured by MRI

#### Volumetric Analysis

Consistent with previous findings (7), total brain volume was decreased in SGA piglets (*p* = 0.041), with no difference between diets. The volumes of all regions of interest (ROI) were decreased in SGA except for the cerebral aqueduct, fourth ventricle, and third ventricle (Supplementary Table S1). Fewer ROI were decreased by SGA when expressed relative to the total brain volume of each piglet (Table 2). HF diet increased the relative volumes of several key ROIs and WM (*p* = 0.003).

**Table 2:** Effects of birth weight and HF on relative brain region volumes (% of whole brain) of 4 week-old piglets^a^.

	**Treatment**				**Pooled SEM**	***P*****-value**^**b**^		
**Region of interest**	**AGA**	**AGA**	**SGA**	**SGA**		**Size**	**Diet**	Size*Diet
	**CON**	**HF**	**CON**	**HF**				
Caudate	0.71	0.68	0.73	0.71	0.04	0.197	0.223	0.706
Cerebellum	8.78	9.02	8.87	8.82	0.27	0.683	0.487	0.286
**Cerebral aqueduct**	0.06	0.07	0.07	0.07	0.01	**0.011**	0.953	0.660
Corpus callosum	0.54	0.55	0.57	0.57	0.02	0.051	0.542	0.729
Fourth ventricle	0.07	0.07	0.07	0.08	0.01	0.066	0.543	0.521
Gray matter	54.69	53.64	55.27	56.06	2.11	0.165	0.905	0.391
Hypothalamus	0.35	0.36	0.37	0.37	0.01	0.050	0.462	0.636
**Internal capsule**	2.09	2.26	2.18	2.30	0.08	0.095	**<0.001**	0.477
Lateral ventricle	0.73	0.64	0.67	0.67	0.06	0.609	0.136	0.147
**Left cortex**	23.58	24.22	24.08	24.88	0.56	**0.046**	**0.015**	0.779
Left hippocampus	0.68	0.68	0.69	0.68	0.02	0.746	0.883	0.435
**Medulla**	2.66	2.75	2.60	2.74	0.09	0.430	**0.014**	0.610
**Midbrain**	2.63	2.77	2.72	2.86	0.07	**0.017**	**<0.001**	0.944
Olfactory bulb	3.42	3.76	3.42	3.46	0.22	0.192	0.092	0.196
**Pons**	1.54	1.60	1.61	1.71	0.06	**0.004**	**0.010**	0.515
**Putamen**	0.56	0.59	0.59	0.62	0.02	**0.001**	**0.004**	0.711
**Right cortex**	24.54	25.10	24.70	25.59	0.65	0.318	**0.033**	0.619
Right hippocampus	0.71	0.71	0.71	0.70	0.02	0.522	0.669	0.537
**Thalamus**	2.28	2.32	2.35	2.43	0.06	**0.010**	0.061	0.496
**Third ventricle**	0.06	0.07	0.07	0.07	0.01	**0.019**	0.315	0.767
**White matter**	24.25	26.78	24.37	25.73	1.19	0.443	**0.003**	0.335

a *Values are means of pigs with MRI data collected at 26–29 days of age. Relative volumes of regions of interest were obtained by determining their percent of whole brain volume for individual pigs prior to statistical analysis. AGA CON: n = 8; AGA HF: n = 8, n = 7 (lateral and fourth ventricle); SGA CON: n = 8, n = 7 (fourth ventricle), n = 6 (lateral ventricle); SGA HF: n = 9, n = 8 (gray matter, lateral ventricle, white matter). Bold values are significance*.

b Size, main effect of birth weight (i.e., AGA vs. SGA); Diet, main effect of dietary intervention (i.e., HF vs. CON); Size*Diet, interaction effect of birth weight, and dietary intervention.

*AGA, appropriate for gestational age; SGA, small for gestational age; CON, control; HF, hydrolyzed fat*.

#### Voxel-Based Morphometry

**Figure 3:**
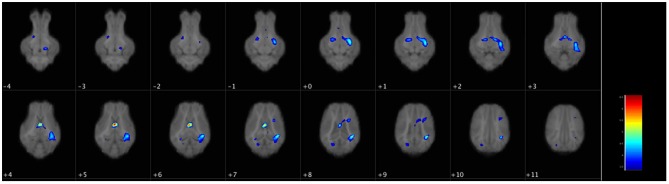
Voxel-based morphometry map of increased white matter clusters in HF piglets compared to CON. Voxel-based morphometry revealed HF fed piglets have more white matter in several key regions compared to CON fed piglets regardless of birth weight. These areas were the next to the lateral ventricle (periventricular white matter), the internal capsule, the left and right cortices, the midbrain, and the hypothalamus. Each image is a 1 mm axial slice composite of all piglet brains. The color bar represents the pseudo-T statistic indicating level of significance, with red indicating highest statistical difference between voxels. Values are 6–8 replicate pigs with MRI data collected at 26–29 days of age as described in Table 3. AGA, appropriate for gestational age; SGA, small for gestational age; CON, control; HF, hydrolyzed fat.

There were no differences in WM volume between SGA and AGA piglets, however, there was a main effect of diet (*p* ≤ 0.001). *T*-tests of dietary effects across birth weights revealed greater WM volumes in the cerebellum of CON fed piglets, while HF fed piglets had more WM adjacent to the lateral ventricle, in the right and left cortices, internal capsule, midbrain, and hypothalamus (Figure 3, Table 3).

**Table 3:** Effects of birth weight and HF on gray matter and white matter differences as determined by voxel-based morphometric analysis.

		**Cluster** **voxels^**a**^**	**Peak-level**	**Local maxima coordinates**		
	**Region of interest**		***P*-value^**b**^**	**x**	**y**	**z**
**White matter**						
CON > HF	Cerebellum	128	< 0.001	−14	−11.2	−4.9
HF > CON	Lateral ventricle	2851	< 0.001	−0.7	11.2	4.9
	Right cortex		< 0.001	13.3	−3.5	9.1
	Internal capsule		< 0.001	11.9	3.5	−0
	Lateral ventricle	33	< 0.001	−10.5	−5.6	6.3
	Midbrain	134	< 0.001	6.3	−2.8	−4.2
	Hypothalamus	51	< 0.001	4.2	8.4	−7.7
	Left cortex	129	< 0.001	−7.7	−11.2	7.7
**Gray matter**						
**CON**						
AGA > SGA	No significant findings					
SGA > AGA	Olfactory bulb	98	< 0.001	5.6	26.6	−7.7
	Right cortex	72	< 0.001	9.1	−5.6	18.9
	Right cortex	65	< 0.001	−14	−11.2	11.9
	Left cortex	23	< 0.001	−5.6	−14	11.2
**HF**						
AGA > SGA	Right cortex	51	< 0.001	3.5	−6.3	-7
SGA > AGA	Right cortex	246	< 0.001	18.9	−0.7	−6.3
	Right cortex	27	< 0.001	−18.2	10.5	5.6
**AGA**						
CON > HF	Olfactory bulb	642	< 0.001	−2.1	24.5	−5.6
	Right cortex	824	< 0.001	2.8	−8.4	12.6
	Left cortex		< 0.001	−7.7	−9.8	16.8
	Left cortex		< 0.001	−2.1	−1.4	15.4
	Lateral ventricle	182	< 0.001	3.5	4.2	−7
	Left cortex	153	< 0.001	−15.4	19.6	8.4
	Left cortex	92	< 0.001	−7.7	−15.4	4.2
	Thalamus	375	< 0.001	−0.7	9.1	0.7
	Right cortex	93	< 0.001	9.1	−9.8	−0.7
	Right cortex	23	0.001	12.6	−17.5	2.8
HF > CON	Olfactory bulb	26	0.001	4.9	26.6	−10.5
**SGA**						
CON > HF	Left cortex	290	< 0.001	−20.3	−4.9	4.2
	Cerebellum	433	< 0.001	−12.6	−9.8	−2.8
	Cerebellum		< 0.001	−14	−18.2	4.2
	Right hippocampus	378	< 0.001	3.5	−5.6	4.9
	Left cortex	32	< 0.001	−8.4	0.7	9.1
	Cerebellum	196	< 0.001	11.2	−11.2	−4.9
	Left cortex	178	< 0.001	−7	−14.7	5.6
	Right cortex	399	< 0.001	11.9	−15.4	6.3
	Lateral ventricle	50	< 0.001	5.6	4.9	8.4
	Left cortex	128	< 0.001	−11.2	20.3	13.3
	Left cortex	32	< 0.001	−12.6	14.7	7.7
HF > CON	No significant findings					

*Tests used a threshold of p = 0.001 uncorrected. AGA CON n = 8, AGA HF n = 8, SGA CON n = 8, SGA HF n = 9*.

a *Clusters > 20 were included in analysis with threshold set at p = 0.001*.

b *Uncorrected P-values*.

*AGA, appropriate for gestational age; SGA, small for gestational age; CON, control; HF, hydrolyzed fat*.

HF AGA piglets had clusters of decreased GM compared to CON AGA piglets in the olfactory bulb, several areas in the left and right cortices, adjacent to the lateral ventricle, and the thalamus. HF AGA piglets had a single area of increased GM in the olfactory bulb compared to CON AGA. Volumetric differences due to SGA were revealed in GM, but not WM (Table 3). Since there was a size*diet interaction (*p* ≤ 0.001) on GM volume, *T*-tests were performed to determine specific effects of SGA and HF diet. CON fed SGA piglets had greater GM volumes in several areas of the left cortex, cerebellum, right hippocampus, right cortex, and adjacent to the lateral ventricle compared to HF fed SGA piglets. There were no ROIs where HF fed SGA piglets had more GM than CON fed SGA piglets. There were areas of differences in GM composition between SGA and AGA piglets on HF diet, localized to the right cortex. CON SGA piglets had areas of increased GM volume in the olfactory bulb and both cortices compared to AGA CON piglets.

#### Diffusion Tensor Imaging

**Figure 4:**
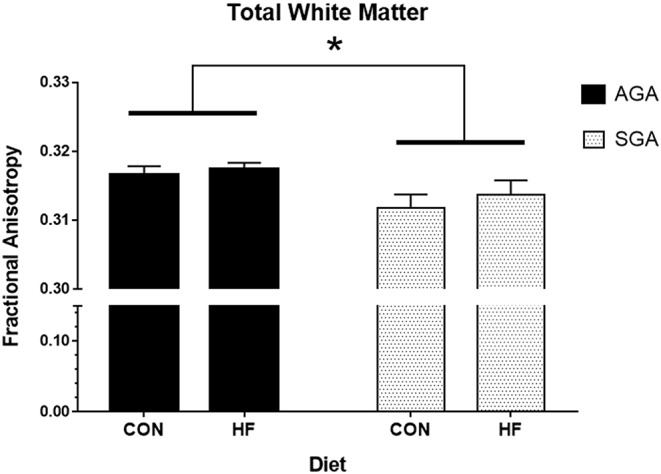
Total white matter fractional anisotropy in AGA and SGA piglets fed HF or CON formula. Fractional anisotropy, a measure of white matter microstructural integrity, was decreased in SGA piglets *p = 0.022. AGA, appropriate for gestational age; SGA, small for gestational age; CON, control; HF, hydrolyzed fat. Data are presented as mean ± SEM, AGA *n* = 5 per group, SGA *n* = 6 per group.

Total WM Fractional Anisotropy (FA) was decreased in SGA piglets compared to AGA piglets (Figure 4, Supplementary Table S2). WM FA was also decreased in SGA piglet right cortex. The corpus callosum of SGA piglets displayed decreased radial diffusivity, axial diffusivity, and mean diffusivity compared to AGA piglets. In addition, the thalamus of SGA piglets had reduced axial diffusivity compared to AGA piglets. HF-fed SGA and AGA piglets had decreased radial diffusivity, axial diffusivity, and mean diffusivity in the corpus callosum (Supplementary Table S2).

#### Single-Voxel Spectroscopy

Total metabolite concentrations of aspartate (Asp), glutathione (GSH), scyllo-inositol (Scyllo), N-acetylaspartate + N-acetylaspartylglutamate (NAA + NAAG), glutamate + glutamine (Glu + Gln), glycerophosphocholine + phosphocholine (GPC + PCh), creatine + phosphocreatine (Cr + PCr), and myo-inositol (Ins) were measured in the hippocampus of AGA and SGA piglets fed CON and HF diets (Table 4). Concentrations of large macromolecules (MM) and lipids (Lip) were measured as well. There were no effects of size, diet, or interactions on the metabolites measured. SGA had an effect on the concentration of MM09 + Lip09, and there was a size*diet interaction on the concentrations of MM14 + Lip13a + Lip13b + MM12. The two numbers in our naming convention refer to the resonant frequency, e.g., “09” corresponds to 0.9 ppm.

**Table 4:** Effects of birth weight and HF on hippocampal metabolism as determined by single-voxel spectroscopy in 4 week-old piglets^a^.

	**Treatment**				**Pooled SEM**	***P*****-Value**^**b**^		
**Metabolite**	**AGA CON**	**AGA HF**	**SGA CON**	**SGA HF**		**Size**	**Diet**	Size*Diet
Asp	2.678	2.728	2.688	2.840	0.243	0.620	0.417	0.679
GSH	2.181	2.330	2.286	2.380	0.215	0.474	0.267	0.803
Ins	7.094	7.322	7.120	7.344	0.261	0.855	0.093	0.990
Scyllo	0.379	0.379	0.374	0.337	0.033	0.173	0.275	0.263
GPC + PCh	1.765	1.814	1.689	1.746	0.106	0.156	0.288	0.939
NAA + NAAG	5.755	5.835	5.745	6.027	0.280	0.521	0.204	0.474
Cr + PCr	4.673	4.720	4.630	4.659	0.169	0.544	0.657	0.916
Glu + Gln	8.368	9.095	8.394	8.662	0.645	0.533	0.134	0.482
MM14 + Lip13a + Lip13b + MM12	10.970	8.716	8.196	9.195	1.206	0.068	0.307	**0.012**
MM09 + Lip09	5.185	4.646	3.943	4.435	0.555	**0.014**	0.933	0.073
MM20 + Lip20	8.972	9.718	8.432	8.868	0.815	0.099	0.157	0.706

a *Values are means of 6–9 replicate pigs with MRI data collected at 26–29 d of age. AGA CON n = 8, n = 7 (MM14 + Lip13a + Lip13b + MM12); AGA HF n = 8, n = 7 (Scyllo), n = 6 (Asp); SGA CON n =8, n = 7 (Asp, Scyllo, MM14 + Lip13a + Lip13b + MM12); SGA HF n = 9. Bold values are significance*.

b Size, main effect of birth weight (i.e., AGA vs. SGA); Diet, main effect of dietary intervention (i.e., HF vs. CON); Size*Diet, interaction effect of birth weight, and dietary intervention.

*Data presented as means and pooled SEM*.

*AGA, appropriate for gestational age; SGA, small for gestational age; CON, control; HF, hydrolyzed fat; NAA + NAAG, N-acetylaspartate + N-acetylaspartylglutamate; GPC + PCh, glycerophosphocholine + phosphocholine; Cr + PCr, creatine + phosphocreatine; Glu + Gln, glutamate + glutamine; MM, macromolecule; Lip, lipid*.

AGA CON piglets had higher concentrations of MM14 + Lip13a + Lip13b + MM12 in their hippocampi compared to SGA CON piglets. There was no difference in MM14 + Lip13a + Lip13b + MM12 concentration between any of the other groups.

### HF Diet Increases ARA Levels in the Hippocampus and Cerebellum

HF diet increased the percentage of total fatty acids (%total) of ARA and 22:5n6, an elongation-desaturation product of ARA, in the cerebellum (Table 5). There were no differences in DHA (%total) between groups, either for the cerebellum or the hippocampus (Tables 5, 6). HF diet significantly decreased 22:5n3, a precursor of DHA in both hippocampus and cerebellum (Tables 5, 6). There was no difference between groups in the total amount of fatty acids in the hippocampus (Supplementary Table S3) or the cerebellum (Supplementary Table S4).

**Table 5:** Fatty acid composition of piglet cerebellum (%)^a^^,^^b^.

	**AGA**		**SGA**		***P*****-value**		
**FAME**	**CON**	**HF**	**CON**	**HF**	**Size**	**Diet**	Size*Diet
14:0	0.45 ± 0.04	0.38 ± 0.03	0.44 ± 0.03	0.40 ± 0.04	0.685	**<0.001**	0.206
16:0 DMA	2.67 ± 0.10	2.62 ± 0.12	2.59 ± 0.16	2.67 ± 0.16	0.737	0.720	0.212
16:0	17.39 ± 0.85	17.60 ± 0.70	17.57 ± 0.89	17.92 ± 0.80	0.375	0.324	0.818
16:1n9	0.47 ± 0.05	0.50 ± 0.07	0.47 ± 0.07	0.50 ± 0.07	0.983	0.279	0.993
16:1n7	0.70 ± 0.05	0.72 ± 0.05	0.70 ± 0.09	0.72 ± 0.07	0.929	0.387	0.845
18:0 DMA	3.81 ± 0.12	3.82 ± 0.14	3.76 ± 0.10	3.75 ± 0.09	0.141	0.947	0.701
18:1 DMA	1.69 ± 0.18	1.61 ± 0.21	1.72 ± 0.26	1.58 ± 0.13	0.991	0.125	0.689
18:0	20.69 ± 0.35	20.86 ± 0.49	20.74 ± 0.79	20.80 ± 0.24	0.982	0.527	0.756
18:1n9	16.74 ± 0.80	16.27 ± 0.71	16.74 ± 1.05	16.24 ± 0.52	0.966	0.088	0.955
18:1n7	4.46 ± 0.29	4.53 ± 0.27	4.30 ± 0.52	4.37 ± 0.26	0.212	0.565	0.979
18:2n6	1.34 ± 0.10	1.26 ± 0.12	1.32 ± 0.15	1.28 ± 0.08	0.607	0.340	0.647
20:0	0.65 ± 0.08	0.61 ± 0.06	0.63 ± 0.09	0.64 ± 0.05	0.930	0.634	0.433
20:1n9	0.98 ± 0.13	0.97 ± 0.14	1.06 ± 0.19	0.92 ± 0.09	0.806	0.143	0.209
20:2n6	0.27 ± 0.04	0.29 ± 0.04	0.27 ± 0.03	0.28 ± 0.04	0.797	0.271	0.960
20:3n6	0.49 ± 0.06	0.45 ± 0.07	0.47 ± 0.05	0.43 ± 0.05	0.228	0.066	0.824
20:4n6	8.53 ± 0.43	8.93 ± 0.45	8.55 ± 0.83	8.95 ± 0.21	0.935	**0.037**	0.997
22:00	0.75 ± 0.17	0.69 ± 0.11	0.70 ± 0.20	0.70 ± 0.10	0.673	0.495	0.546
22:1n9	0.24 ± 0.07	0.21 ± 0.04	0.22 ± 0.09	0.21 ± 0.04	0.708	0.452	0.708
22:2n6	0.21 ± 0.04	0.21 ± 0.02	0.22 ± 0.05	0.22 ± 0.03	0.614	0.729	0.825
22:4n6	3.27 ± 0.23	3.37 ± 0.24	3.27 ± 0.17	3.40 ± 0.15	0.830	0.087	0.817
22:5n6	1.42 ± 0.22	1.67 ± 0.22	1.43 ± 0.21	1.81 ± 0.26	0.329	**<0.001**	0.407
22:5n3	0.31 ± 0.04	0.27 ± 0.05	0.32 ± 0.05	0.29 ± 0.05	0.291	**0.035**	0.565
24:0	0.88 ± 0.27	0.81 ± 0.19	0.79 ± 0.24	0.82 ± 0.12	0.565	0.825	0.482
22:6n3	7.60 ± 0.66	7.49 ± 0.37	7.81 ± 0.59	7.31 ± 0.41	0.905	0.097	0.279
24:1n9	1.66 ± 0.48	1.62 ± 0.39	1.63 ± 0.52	1.62 ± 0.26	0.916	0.857	0.932
TUFA	2.35 ± 0.35	2.24 ± 0.29	2.29 ± 0.47	2.16 ± 0.21	0.587	0.324	0.923

a *Values presented as the means ± SEM of percent of total fatty acids of 8–9 replicate pigs collected at 26–29 days of age. AGA CON n = 8, AGA HF n = 8, SGA CON n = 9, SGA HF n = 9. Bold values are significance*.

b Size, main effect of birth weight (i.e., AGA vs. SGA); Diet, main effect of dietary intervention (i.e., HF vs. CON); Size*Diet, interaction effect of birth weight, and dietary intervention.

*AGA, appropriate for gestational age; SGA, small for gestational age; CON, control; HF, hydrolyzed fat; FAME, fatty acid methyl ester; DMA, dimethylacetal; TUFA, total unidentified fatty acids*.

**Table 6:** Fatty acid composition of piglet hippocampus (%)^a^^,^^b^.

	**AGA**		**SGA**		***P*****-value**		
**FAME**	**CON**	**HF**	**CON**	**HF**	**Size**	**Diet**	Size*Diet
14:0	0.53 ± 0.02	0.47 ± 0.03	0.54 ± 0.03	0.47 ± 0.04	0.818	**<0.001**	0.630
16:0 DMA	2.64 ± 0.19	2.60 ± 0.19	2.56 ± 0.19	2.72 ± 0.23	0.790	0.393	0.153
16:0	16.77 ± 0.53	16.95 ± 0.87	17.04 ± 0.72	16.99 ± 0.60	0.518	0.778	0.625
16:1n9	0.49 ± 0.03	0.50 ± 0.08	0.50 ± 0.05	0.48 ± 0.06	0.936	0.868	0.468
16:1n7	0.88 ± 0.07	0.85 ± 0.09	0.92 ± 0.10	0.91 ± 0.09	0.129	0.461	0.843
18:0 DMA	4.10 ± 0.18	4.16 ± 0.12	4.11 ± 0.14	4.03 ± 0.15	0.249	0.885	0.167
18:1 DMA	1.54 ± 0.19	1.51 ± 0.27	1.53 ± 0.22	1.59 ± 0.25	0.690	0.860	0.618
18:0	22.00 ± 0.54	21.92 ± 0.57	22.07 ± 0.61	21.51 ± 0.88	0.465	0.179	0.295
18:1n9	15.70 ± 0.92	15.33 ± 1.06	15.35 ± 1.28	16.12 ± 1.40	0.591	0.628	0.172
18:1n7	4.66 ± 0.40	4.81 ± 0.27	4.59 ± 0.37	4.60 ± 0.31	0.227	0.508	0.546
18:2n6	1.26 ± 0.10	1.17 ± 0.08	1.26 ± 0.13	1.23 ± 0.10	0.466	0.101	0.469
20:0	0.52 ± 0.04	0.53 ± 0.03	0.53 ± 0.05	0.56 ± 0.07	0.331	0.260	0.430
20:1n9	0.59 ± 0.08	0.59 ± 0.07	0.59 ± 0.07	0.66 ± 0.16	0.301	0.311	0.282
20:2n6	0.32 ± 0.05	0.33 ± 0.06	0.31 ± 0.05	0.34 ± 0.06	0.984	0.291	0.572
20:3n6	0.51 ± 0.06	0.45 ± 0.04	0.48 ± 0.07	0.45 ± 0.03	0.406	**0.014**	0.505
20:4n6	8.84 ± 0.37	9.09 ± 0.38	8.87 ± 0.47	8.77 ± 0.48	0.341	0.612	0.258
22:0	0.81 ± 0.10	0.79 ± 0.13	0.80 ± 0.15	0.84 ± 0.15	0.716	0.776	0.526
22:1n9	0.29 ± 0.06	0.29 ± 0.08	0.27 ± 0.07	0.30 ± 0.08	0.870	0.404	0.491
22:2n6	0.36 ± 0.09	0.36 ± 0.09	0.36 ± 0.10	0.37 ± 0.08	0.801	0.784	0.814
22:4n6	4.17 ± 0.27	4.27 ± 0.21	4.26 ± 0.19	4.02 ± 0.0.42	0.418	0.521	0.106
22:5n6	1.88 ± 0.15	2.00 ± 0.37	1.99 ± 0.38	2.11 ± 0.52	0.428	0.361	0.976
22:5n3	0.21 ± 0.02	0.17 ± 0.03	0.23 ± 0.02	0.19 ± 0.03	**0.005**	**<0.001**	0.838
24:0	0.99 ± 0.20	1.01 ± 0.24	1.02 ± 0.29	1.08 ± 0.24	0.579	0.694	0.792
22:6n3	6.04 ± 0.82	5.88 ± 0.92	6.03 ± 0.96	5.59 ± 1.04	0.644	0.361	0.668
24:1n9	1.48 ± 0.24	1.57 ± 0.34	1.52 ± 0.29	1.61 ± 0.25	0.695	0.370	0.986
TUFA	2.42 ± 0.30	2.39 ± 0.37	2.29 ± 0.39	2.47 ± 0.43	0.821	0.579	0.419

a *Values presented as the means ± SEM of percent of total fatty acids of 8–9 replicate pigs collected at 26–29 days of age. AGA CON n = 8, AGA HF n = 8, SGA CON n = 9, SGA HF n = 9. Bold values are significance*.

b Size, main effect of birth weight (i.e., AGA vs. SGA); Diet, main effect of dietary intervention (i.e., HF vs. CON); Size*Diet, interaction effect of birth weight, and dietary intervention.

*AGA, appropriate for gestational age; SGA, small for gestational age; CON, control; HF, hydrolyzed fat; FAME, fatty acid methyl ester; DMA, dimethylacetal; TUFA, total unidentified fatty acids*.

### HF Diet and SGA Status Have Unique Impacts on the Hippocampal Transcriptome

**Figure 5:**
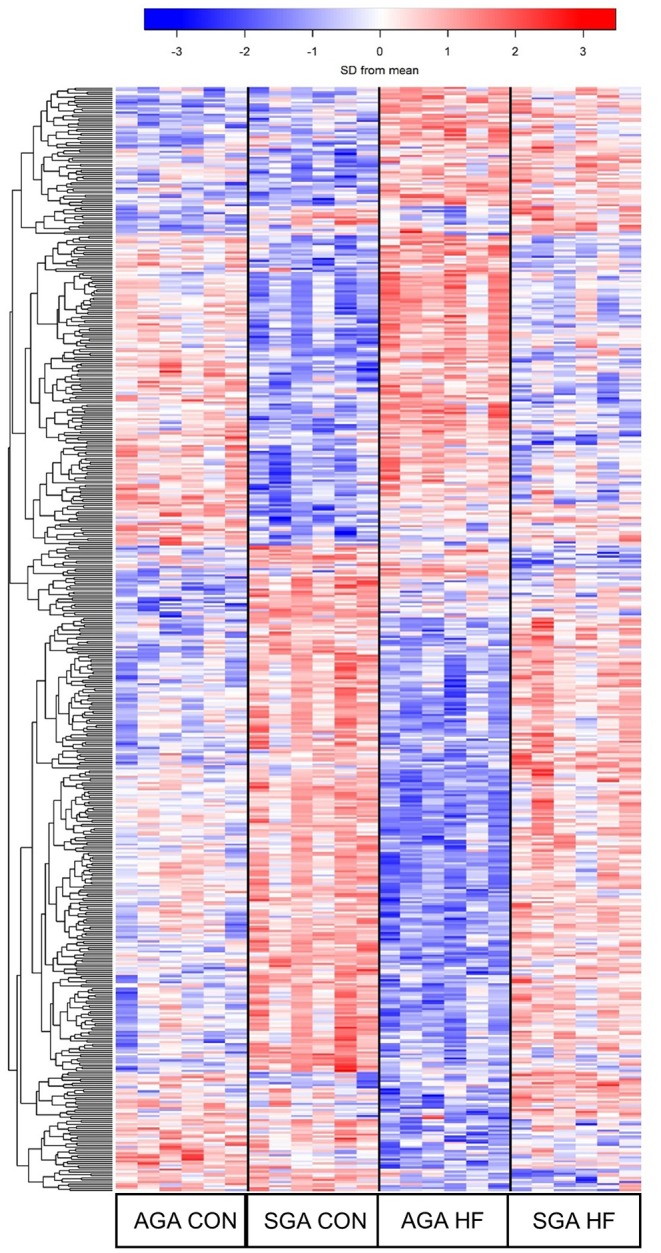
Heat map demonstrating the unique gene expression patterns in the hippocampi due to SGA and HF diet. Each row represents a gene and its relative expression (blue = down-regulation; red= up-regulation) in each piglet. 319 DEGs were identified in response to SGA, 105 DEGs were identified in response to diet, and 24 DEGs were involved in a SGA by diet interaction. AGA CON *n* = 6, AGA HF *n* = 6, SGA CON *n* = 6, SGA HF *n* = 6. AGA, appropriate for gestational age; SGA, small for gestational age; CON, control; HF, hydrolyzed fat; DEG, differentially expressed genes.

The hierarchical clustering heat map of the overall DEG patterns revealed main effects of SGA and diet on hippocampal transcriptomic profiles (Figure 5). A total of 319 DEGs were identified in response to SGA, of which 206 were up-regulated and 113 were down-regulated. A total of 105 DEGs were identified in response to HF diet, of which 59 were up-regulated and 46 down-regulated. A size*diet interaction affected 24 genes, with 7 genes down-regulated and 17 genes up-regulated. Pair-wise comparisons of the treatments revealed differences between SGA piglets and AGA piglets on CON diet, with 42 up-regulated genes and 51 down-regulated genes. SGA HF piglets compared to AGA HF piglets revealed 130 up-regulated and 29 down-regulated genes. The HF diet compared to CON diet led to 22 up-regulated genes in AGA piglets and 40 down-regulated genes. For SGA piglets, the HF diet compared to CON diet led to 33 up-regulated genes and 15 down-regulated genes.

The GO categories enriched for up-regulated DEGs in SGA piglets were GO Biological Processes “cellular sodium ion homeostasis” (*p* = 0.011) and “ATP hydrolysis coupled proton transport” (*p* = 0.040), and GO Molecular Functions “protein tyrosine phosphatase activity” (*p* = 0.18). The full list of KEGG pathways related to up-regulated DEGs in SGA hippocampus is provided (Supplementary Table S5). Of the 46 KEGG pathways identified with an enrichment score >1.3, we focused on 22 that we felt had high relevance to expression pathways in the developing brain (Table 7). The GO categories enriched for down-regulated DEGs in SGA piglets were the GO Biological Processes “translation” (*p* < 0.001) and “cell division” (*p* = 0.039) and GO Molecular Functions “structural constituent of ribosome” (*p* < 0.001). One KEGG pathway associated with down-regulated DEGs, “ribosome,” was enriched (*p* < 0.001) due to SGA.

**Table 7:** Pathway analysis of select up-regulated genes due to SGA in the hippocampus of 2 week-old piglets^a^.

**Top enriched KEGG pathways**	**Enrichment score**	**Gene count**	***P*-Value**	**Benjamini value^**b**^**
cAMP signaling pathway	1.89	8	8.20E-03	9.10E-02
Calcium signaling pathway	1.64	9	1.10E-03	2.70E-02
Circadian entrainment	1.64	7	1.40E-03	2.90E-02
Olfactory transduction	1.64	4	2.40E-03	4.00E-02
Oxytocin signaling pathway	1.64	8	3.70E-03	5.40E-02
Amphetamine addiction	1.64	5	8.10E-03	9.60E-02
Long-term potentiation	1.64	5	8.60E-03	8.80E-02
Cholinergic synapse	1.64	6	1.30E-02	1.20E-01
Dopaminergic synapse	1.64	6	1.70E-02	1.40E-01
Inflammatory mediator regulation of TRP channels	1.64	5	2.90E-02	2.00E-01
Retrograde endocannabinoid signaling	1.64	5	3.30E-02	2.10E-01
Glioma	1.64	4	4.10E-02	2.40E-01
Glutamatergic synapse	1.64	5	6.40E-02	3.00E-01
Wnt signaling pathway	1.64	5	7.00E-02	3.10E-01
GnRH signaling pathway	1.64	4	8.70E-02	3.60E-01
Neurotrophin signaling pathway	1.64	4	1.90E-01	5.60E-01
GABAergic synapse	1.64	3	2.70E-01	6.70E-01
ErbB signaling pathway	1.64	3	3.00E-01	6.90E-01
Morphine addiction	1.64	3	3.00E-01	6.90E-01
HIF-1 signaling pathway	1.64	3	3.50E-01	7.40E-01
Serotonergic synapse	1.64	3	3.70E-01	7.50E-01

a *Enrichment scores as determined by Functional Annotation Clustering in DAVID v.6.8, which rank overall importance of the annotation term groups. The most relevant 22 are displayed, see Supplementary Table S1 for complete list. AGA CON n = 6, AGA HF n = 6, SGA CON n = 6, SGA HF n = 6*.

b *Main effect of birth weight (i.e., SGA vs. AGA); the Benjamini value corrects for multiple comparisons*.

*AGA, appropriate for gestational age; SGA, small for gestational age*.

Although there was a main effect of diet on the transcription profile of the hippocampus, the 59 genes that were up-regulated by HF diet did not have gene enrichment >1.3. For down-regulated DEGs, however, the GO Biological Process enriched in HF piglets was “immune response” (*p* = 0.033), with enriched genes matching 10 KEGG pathways (Table 8). The size*diet interaction did not have gene enrichment that could be mapped to any clusters or pathways.

**Table 8:** Pathway analysis of down-regulated genes due to HF diet in the hippocampus of 2 week-old piglets^a^.

**Top enriched KEGG pathways**	**Enrichment score**	**Gene count**	***P*-Value**	**Benjamini value^**b**^**
Herpes simplex infection	2.3	6	4.40E-06	2.90E-04
Viral myocarditis	2.3	4	1.00E-04	3.40E-03
Graft-versus-host disease	2.3	3	7.10E-04	1.60E-02
Allograft rejection	2.3	3	8.60E-04	1.40E-02
Autoimmune thyroid disease	2.3	3	9.40E-04	1.20E-02
Type I diabetes mellitus	2.3	3	1.20E-03	1.30E-02
Antigen processing and presentation	2.3	3	4.60E-03	4.30E-02
HTLV-1 infection	2.3	4	7.90E-03	6.40E-02
Cell adhesion molecules (CAMs)	2.3	3	1.80E-02	1.30E-01
Phagosome	2.3	3	2.30E-02	1.50E-01

a *Enrichment scores as determined by Functional Annotation Clustering in DAVID v.6.8, which rank overall importance of the annotation term groups. AGA CON n = 6, AGA HF n = 6, SGA CON n = 6, SGA HF n = 6*.

b *Main effect of diet (i.e., CON vs. HF); the Benjamini value corrects for multiple comparisons*.

*CON, control; HF, hydrolyzed fat*.

### HF Diet Affects Performance in a Cognitive Task

**Figure 6:**
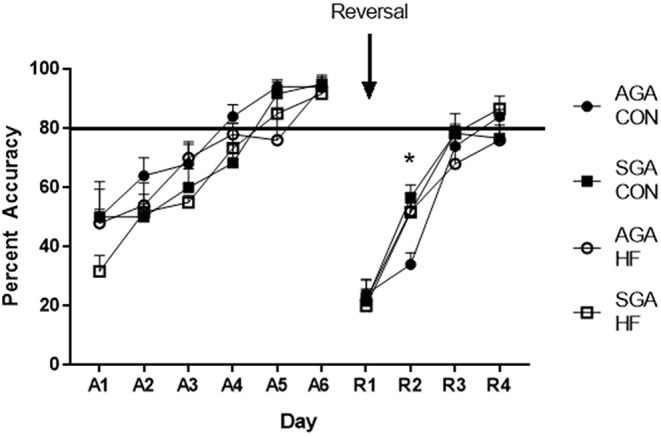
Accuracy of SGA and AGA piglets fed CON or HF formula in a spatial T-maze task. Performance of all groups of piglets during both the acquisition (A1–6) phase and the reversal (R1–4) phase of the T-maze task improved over time (*p* <0.001). There was no treatment effects during acquisition. There was a diet*day*size interaction during reversal (*p* = 0.026), due to AGA CON piglets lagging behind on R2 (*p* = 0.017). *p < 0.05. AGA, appropriate for gestational age; SGA, small for gestational age; CON, control; HF, hydrolyzed fat. Data are presented as mean ± SEM, AGA CON *n* = 5, AGA HF *n* = 5, SGA CON *n* = 6, SGA HF *n* = 6.

During the acquisition phase of the test, piglet performance improved over time (*p* < 0.001. Figure 6) and all groups reached criterion by day 5, demonstrating that they learned the task. There was no effect of diet (*p* = 0.13) or size (*p* = 0.08) on piglet performance. There were no interactions.

There was an interaction of size*diet *day (*p* = 0.026) during reversal. All groups improved over time (*p* < 0.001; Figure 6). Slicing the 3-way interaction by day revealed that on reversal day 2 (R2), AGA CON piglets had lower accuracy compared to AGA HF piglets (*p* = 0.020), SGA CON piglets (*p* = 0.003), and SGA HF piglets (*p* = 0.018). There was no difference in performance between the groups on any other day of reversal (R1 *p* = 0.96, R3 *p* = 0.45, R4 *p* = 0.35).

## Discussion

During IUGR, affected fetuses undergo changes in circulation that redirect blood supply to the brain, known as the “brain-sparing effect.” The brain-sparing effect was thought to be an adaptation which preserved brain function at the cost of the rest of the body, but may actually be a maladaptive consequence of peripheral vascular congestion (34). The brain-sparing effect in IUGR infants has been correlated to worse neurodevelopmental outcomes including difficulties with creative problem solving, attention and executive functions, and visuomotor organization (35, 36). Brain to body weight ratios of SGA piglets in this study suggested that the brain-sparing effect had occurred. HF diet did not have an effect on brain weight or body weight in AGA or SGA piglets.

Infants born either premature or SGA due to IUGR have lower levels of DHA in cortical structures as maternal circulation becomes disrupted during the time when DHA uptake into the brain increases (37–39). We hypothesized that the HF formula diet would increase fatty acid availability and uptake into the brain, as the HF formula contains soy lecithin– a source of phospholipids such as phosphatidylcholine, phosphatidylethanolamine, and phosphatidylinositol (40). Phospholipids play an important role in brain development and myelination. A previous study showed that neonatal piglets supplemented with dietary phospholipids had increased brain weight, gray matter, and white matter volume compared to unsupplemented piglets (41). In addition, phospholipids serve as carriers of long chain polyunsaturated fatty acids (LC-PUFA) such as ARA and DHA, and lysophosphotidylcholine can facilitate LC-PUFA entry into the brain [reviewed in (42)]. In our experiment, fatty acid analysis revealed that HF fed piglets had increased levels of ARA in the hippocampus and cerebellum, potentially due to increased absorption and/or transport of ARA conjugated to PC or LPC lecithin or soybean free fatty acids and their subsequent conversion to ARA. Although ARA is generally associated with pro-inflammatory processes such as prostaglandin production, it has been shown to influence myelin producing oligodendrocytes depending on the microenvironmental context (43). ARA and one of its metabolites, 15(*S*)-hydroxyeicosatetraenoic acid [15(*S*)-HETE], activate the nuclear receptor peroxisome proliferator-activated receptor-γ (PPARγ), which has anti-inflammatory benefits and provides neuroprotection in a rat model of intracerebral hemorrhage (44). Since immune response gene expression pathways were down-regulated in the hippocampus of HF fed piglets, it is possible that increased ARA levels are mediating a more neurosupportive environment as opposed to neurotoxic. It is, however, important to do further studies to ensure that detrimentally high levels do not occur. HF diet did not increase the total amount of fatty acids in the cerebellum or hippocampus, nor did it change DHA levels. SGA status also did not affect DHA levels at 28 days old. It is possible that other brain regions, such as the internal capsule, midbrain, pons, or thalamus, may have altered DHA levels, however, it was not practical to test all regions.

MRI was performed to determine if HF diet would enhance brain growth or microstructure in SGA piglets. There were several brain regions in the SGA piglets likely affected by the brain-sparing effect, including the midbrain, pons, and thalamus, causing them to take up a larger percentage of total brain volume. Human IUGR fetuses also have increased relative thalamus volume, likely due to regional differences in susceptibility to insults and changes in blood flow that occur during IUGR (45). Our results therefore could be explained by increased regional blood flow during brain-sparing, which first occurs in the frontal lobe, then subsequently decreases as IUGR progresses followed by increased blood flow to the basal ganglia which includes the putamen (34, 45). Following patterns described previously (7), SGA piglets in our experiment had smaller absolute volumes of most brain regions, with increases in some relative volumes when normalized as described above. HF diet only affected the absolute volume of one region, the caudate nucleus, and increased the relative volumes of both cortices, internal capsule, medulla, midbrain, pons, putamen, and white matter in both SGA and AGA piglets. Therefore, while IUGR and HF diet both increased relative volume of ROIs, the mechanism by which this occurs is likely different, with IUGR associated with maladaptive redistributed blood flow and HF diet likely due to increased WM volume.

DTI, which measures the restrictions of water diffusion in the brain to determine microstructural tissue integrity (46), revealed decreased total WM integrity (FA) in SGA piglets. Radlowski et al. (7), also found decreased global FA in SGA piglets, suggesting widespread myelination defects in the SGA brain. WM primarily consists of myelinated axons and oligodendrocytes—cells that produce the lipid rich myelin, which insulates axons and improves electrical signals conduction. WM tract deficits due to SGA have been seen in several human studies as well (47–50). Decreased WM tract development is generally associated with reduced IQ (51, 52). In our study, there were no interactions of SGA and HF diet, therefore HF diet did not correct the deficits in WM microstructure in the SGA piglet brain. This finding may be due to the fact that the cortical structural changes caused by IUGR begin *in utero* [reviewed in (53)]; therefore it is unlikely that post-natal dietary intervention can alter these maladaptive structural changes after they have occurred. However, HF increased the total relative volume of WM as well as WM associated structures in both AGA and SGA piglets.

VBM analysis, which compares WM and GM composition between treatment groups, also revealed consistent effects of dietary treatment on WM volumes in both SGA and AGA piglets. HF fed piglets had greater WM volumes in several key structures such as the internal capsule. These findings are particularly interesting since the internal capsule was an area with decreased WM in SGA piglets in a previous study (7). The internal capsule consists of WM tracts that connect the cerebral cortex to lower motor neurons in the spinal cord. Deficits in this area are associated with poor motor control and abnormal gait in low birth weight infants (54).

RNA-sequencing was used to assess the effects of IUGR and HF diet on gene expression patterns in the hippocampus. Currently, there is very limited information about IUGR-related gene expression patterns in the hippocampus, therefore we hoped to provide some mechanisms for the learning difficulties seen in IUGR infants (7, 55) and if HF diet would correct them. Gene ontology and pathway analysis of up-regulated genes in the SGA hippocampus included protein tyrosine phosphatase activity, with half of the KEGG pathways directly related to brain development. Protein tyrosine phosphatases interact with cadherin-catenin complexes, which are essential for proper function of synapses (56). PTP1B is a protein tyrosine phosphatase that has been shown to play a role in normal function of dendritic spines, and knocking out PTP1B in the hippocampus of mice caused morphologically “immature” brains (57). Similar results are seen with other protein tyrosine phosphatases, suggesting they play an important role in modulating learning and memory (57, 58). Increased transcription of these pathways could indicate a breakdown of regulation due to IUGR, possibly affecting learning capability. Analysis of KEGG pathways in the SGA piglet hippocampi revealed upregulation of pathways involved in cAMP signaling, calcium signaling, long-term potentiation, and multiple types of synapses. cAMP signaling regulates many downstream biological processes such as Na^+^/Ca2^+^ influx through NMDA receptors and hyperexcitability in hippocampal neurons (59–61). Schober et al. found NMDA receptor subunit composition was affected by IUGR, which could be associated with synaptic hyperexcitability (62). Increased sodium ion flux through NMDA receptors is also associated with brain injury and neurotoxicity [reviewed by (63)]. These results improve understanding of the increased risk of mental illness in low birth weight infants (64). Along with the upregulation of multiple pathways, there was downregulation of genes associated with ribosomal subunits and cell division. This suggests altered protein translation in the hippocampus of IUGR infants. Combined with altered synaptic gene expression, these expression patterns may be associated with brain structure and functional differences observed in other studies of IUGR neonates (62, 65, 66).

HF diet did not correct altered gene expression patterns observed in the SGA hippocampus. It did, however, decrease genes associated with viral infection and autoimmunity– largely in the phagosome pathway. This is noteworthy because microglia, the resident brain macrophage, phagocytize unwanted neural connections (synaptic pruning) as a normal part of perinatal brain development (67). A recent study by Wlodarczyk et al. (68) examined the transcriptome of neonatal microglia and found downregulation of genes within the GO Term “immune response,” which could suggest a neurosupportive microglial phenotype that promotes myelination. It would therefore be interesting to pursue further studies of the effects of HF diet on neonatal microglial phenotypes.

To assess functional cognitive outcome, piglets underwent testing in the T-maze learning and memory task to determine if the HF dietary intervention could ameliorate the deficits in learning and memory seen by Radlowski et al. (7) or improve overall performance. The task is designed to assess function of the hippocampus, the brain region that plays a pivotal role in spatial learning and memory (29). In the present study, there were no effects of SGA or dietary intervention on the ability of the piglets to acquire the task. During the reversal phase, where the piglets must relearn the location of their individual reward bowl, AGA CON piglets lagged behind the SGA and HF groups on day 2 but caught up on day 3 with no difference in performance between the groups from that point. The reversal phase is a more sensitive measure of the animals' learning ability, as it requires them to unlearn the previously rewarded location and relearn the new one (29, 69, 70). The reversal phase is a widely used measure of cognitive flexibility, which involves several brain regions including the striatum, amygdala, medial prefrontal cortex (mPFC) and the orbitofrontal cortex (OFC), in addition to the hippocampus (71). HF diet may contribute to changes in these regions leading to improved reversal learning. Both the left cortex and right cortex along with the putamen, a component of the striatum, displayed increased relative brain volume as a result of HF diet. Additionally, VBM analysis showed increased white matter as an effect of HF diet in both the left and right cortex. It is possible that development of areas such as the OFC and mPFC were improved by HF diet—for example, improved myelination of OFC projections to the striatum. A limitation of this study, however, is that the pig MRI paradigm used does not afford the resolution to evaluate cortical regions at the level of detail needed to elucidate the exact mechanism, but this is a question to explore further in future experiments.

## Conclusion

This study is novel in that it assessed the effects of hydrolyzed fat diet on brain structure and development in an understudied but vulnerable neonatal population. We find in this study that HF diet did not affect body weight, brain weight, or brain to body weight ratio in AGA or SGA piglets. Based on DTI analysis, HF did not ameliorate the total WM microstructural deficits observed in SGA piglets. HF diet did increase the relative volume of WM and several WM tract-associated regions in both SGA and AGA piglets. HF decreased expression of genes associated with immune response in the hippocampus of both AGA and SGA piglets, but did not correct aberrant gene expression observed in the hippocampus of SGA piglets. When observing possible functional cognitive outcomes of the dietary intervention, we found that HF diet improved accuracy for AGA piglets in the spatial learning and memory T-maze task during the reversal phase. Overall, HF may help enhance WM development in the neonatal brain; however, more studies are needed to assess brain development long-term along with more tests to assess functional cognitive outcomes.

## Data Availability Statement

The data sets generated for this study can be found in NCBI GEO GSE134739 (https://www.ncbi.nlm.nih.gov/geo/query/acc.cgi?acc=GSE134739).

## Ethics Statement

All animal care and experimental procedures were in accordance with the National Research Council Guide for the Care and Use of Laboratory Animals and approved by the University of Illinois at Urbana-Champaign Institutional Animal Care and Use Committee.

## Author Contributions

RJ and ER designed the study. MC, ER, and JW conducted research. MC, MN, JH, BL, JD, RL, and BS analyzed data. MC, JW, JD, BL, RL, BS, TD, and MK wrote the paper. RJ had primary responsibility for final content. All authors read and approved the final manuscript.

### Conflict of Interest

The authors declare that the research was conducted in the absence of any commercial or financial relationships that could be construed as a potential conflict of interest.

## Abbreviations

Asp: aspartate
AGA: appropriate for gestational age
ARA: arachidonic acid
CON: control
DEG: differentially expressed genes
DHA: docosahexaenoic acid
DTI: diffusion tensor imaging
FA: fractional anisotropy
FDR: false discovery rate
FSL: FMRIB Software Library
Gln: gluatamine
Glu: glutamate
GM: gray matter
GPC: glycerophosphocholine
GSH: glutathione
HF: hydrolyzed fat
HOSO: high oleic safflower oil
Ins: myo-inositol
IUGR: intrauterine growth restriction
LC-PUFA: long chain polyunsaturated fatty acids
Lip: lipids
MM: macromolecules
MRI: magnetic resonance imaging
NAA: N-acetylaspartate
NAAG: N-acetylaspartylglutamate
PCr: phosphocreatine
PRESS: Point RESolved Spectroscopy
ROI: region of interest
RQN: RNA quality number
Scyllo: scyllo-inositol
SGA: small for gestational age
sv: surrogate variable
SVS: single voxel spectroscopy
TKX: telazol:ketamine:xylazine
TMM: trimmed mean of M-values
VBM: voxel based morphometry
WM: white matter.
