# Supplementary material for: Hydrolyzed Fat Formula Increases Brain White Matter in Small for Gestational Age and Appropriate for Gestational Age Neonatal Piglets
Source: Front Pediatr. 2020 Feb 12;8:32. doi: 10.3389/fped.2020.00032 (PMC7029735; doi:10.3389/fped.2020.00032)
Supplement: Supplementary file 1 [file Table_1.DOCX]

Supplementary Material

| **Supplementary Table 1.** Effects of birth weight and HF on absolute brain region volumes in mm^3^ of 4-wk-old piglets^1^ | | | | | | | | |
| --- | --- | --- | --- | --- | --- | --- | --- | --- |
|  | Treatment | | | | Pooled SEM | P – value^2^ | | |
| Region of Interest | AGA CON | AGA  HF | SGA CON | SGA HF |  | Size | Diet | Size*Diet |
|  |  |  |  |  |  |  |  |  |
| Caudate | 522.6 | 484.1 | 468.6 | 446.2 | 29.5 | **0.004** | **0.048** | 0.587 |
| Cerebellum | 6471.3 | 6434.8 | 5757.9 | 5568.5 | 354.4 | **<0.001** | 0.529 | 0.670 |
| Cerebral Aqueduct | 46.6 | 46.0 | 47.8 | 46.0 | 3.5 | 0.733 | 0.506 | 0.729 |
| Corpus Callosum | 397.2 | 391.6 | 368.4 | 358.1 | 23.1 | **0.011** | 0.495 | 0.840 |
| Fourth Ventricle | 50.7 | 49.9 | 47.2 | 47.9 | 3.1 | 0.088 | 0.947 | 0.639 |
| Grey Matter | 40341.5 | 38277.6 | 35985.0 | 35048.2 | 2511.5 | **0.005** | 0.242 | 0.657 |
| Hypothalamus | 261.8 | 254.3 | 237.1 | 235.7 | 15.0 | **0.008** | 0.559 | 0.691 |
| Internal Capsule | 1539.3 | 1614.8 | 1420.6 | 1454.9 | 104.6 | **0.013** | 0.303 | 0.697 |
| Lateral Ventricle | 539.2 | 460.1 | 448.5 | 416.1 | 45.6 | **0.007** | **0.022** | 0.157 |
| Left Cortex | 17385.8 | 17271.9 | 15662.3 | 15664.9 | 946.0 | **0.001** | 0.907 | 0.903 |
| Left Hippocampus | 500.3 | 488.8 | 446.7 | 427.9 | 28.7 | **<0.001** | 0.301 | 0.802 |
| Medulla | 1960.4 | 1963.4 | 1690.0 | 1730.4 | 114.5 | **<0.001** | 0.708 | 0.747 |
| Midbrain | 1939.2 | 1972.3 | 1762.9 | 1799.8 | 102.2 | **0.002** | 0.499 | 0.970 |
| Olfactory Bulb | 2524.0 | 2671.5 | 2218.4 | 2187.1 | 187.6 | **<0.001** | 0.540 | 0.348 |
| Pons | 1135.6 | 1141.5 | 1043.7 | 1075.6 | 54.3 | **0.007** | 0.492 | 0.636 |
| Putamen | 411.8 | 419.8 | 385.1 | 388.9 | 26.7 | **0.040** | 0.661 | 0.876 |
| Right Cortex | 18101.5 | 17905.0 | 16082.4 | 16106.6 | 1019.9 | **<0.001** | 0.867 | 0.830 |
| Right Hippocampus | 521.4 | 505.4 | 458.5 | 438.5 | 29.3 | **<0.001** | 0.228 | 0.892 |
| Thalamus | 1685.3 | 1656.3 | 1525.4 | 1530.0 | 83.6 | **0.002** | 0.773 | 0.690 |
| Third Ventricle | 47.6 | 48.7 | 46.9 | 47.2 | 3.2 | 0.492 | 0.663 | 0.807 |
| White Matter | 17904.7 | 19111.4 | 15894.1 | 16159.5 | 1445.8 | **0.002** | 0.317 | 0.520 |
| Whole Brain | 73806.2 | 71335.3 | 64942.3 | 63026.5 | 3658.8 | **0.041** | 0.081 | 0.592 |

^1^Values are means of 7-9 replicate pigs with MRI data collected at 26-29 d of age. Volumes are in units of mm^3^.

^2^Size, main effect of birth weight (i.e. AGA vs. SGA); Diet, main effect of dietary intervention (i.e. HF vs. CON); Size*Diet, interaction effect of birth weight and dietary intervention.

Abbreviations: AGA, appropriate for gestational age; SGA, small for gestational age; CON, control; HF, hydrolyzed fat
