# Supplementary material for: Hydrolyzed Fat Formula Increases Brain White Matter in Small for Gestational Age and Appropriate for Gestational Age Neonatal Piglets
Source: Front Pediatr. 2020 Feb 12;8:32. doi: 10.3389/fped.2020.00032 (PMC7029735; doi:10.3389/fped.2020.00032)
Supplement: Supplementary file 2 [file Table_2.DOCX]

Supplementary Material

| **Supplementary Table 2.** Effects of birth weight and HF on white matter maturation as determined by diffusion tensor imaging in 4-wk-old piglets^1^ | | | | | | | | |
| --- | --- | --- | --- | --- | --- | --- | --- | --- |
|  | Treatment | | | | Pooled SEM | P – Value^2^ | | |
| Region of Interest | AGA CON | AGA HF | SGA CON | SGA HF |  | Size | Diet | Size*Diet |
| **Fractional Anisotropy** |  |  |  |  |  |  |  |  |
| Caudate | 3.27E-01 | 3.26E-01 | 3.27E-01 | 3.17E-01 | 1.01E-02 | 0.367 | 0.315 | 0.407 |
| Corpus Callosum | 2.96E-01 | 2.93E-01 | 2.83E-01 | 2.94E-01 | 1.18E-02 | 0.353 | 0.493 | 0.241 |
| Cerebellum | 1.87E-01 | 1.86E-01 | 1.83E-01 | 1.78E-01 | 7.56E-03 | 0.114 | 0.487 | 0.541 |
| Left Hippocampus | 2.95E-01 | 2.86E-01 | 2.87E-01 | 2.92E-01 | 1.32E-02 | 0.921 | 0.790 | 0.304 |
| Right Hippocampus | 2.89E-01 | 2.81E-01 | 2.86E-01 | 2.86E-01 | 1.12E-02 | 0.935 | 0.503 | 0.448 |
| Internal Capsule | 4.21E-01 | 4.17E-01 | 4.08E-01 | 4.14E-01 | 1.13E-02 | 0.164 | 0.839 | 0.378 |
| Left Cortex | 3.15E-01 | 3.16E-01 | 3.13E-01 | 3.14E-01 | 4.50E-03 | 0.341 | 0.851 | 0.917 |
| Right Cortex | 3.19E-01 | 3.20E-01 | 3.11E-01 | 3.17E-01 | 5.11E-03 | **0.042** | 0.259 | 0.329 |
| Thalamus | 3.10E-01 | 3.13E-01 | 3.06E-01 | 2.97E-01 | 1.24E-02 | 0.135 | 0.668 | 0.380 |
| Total White Matter | 3.16E-01 | 3.17E-01 | 3.12E-01 | 3.14E-01 | 3.41E-03 | **0.022** | 0.426 | 0.767 |
| Whole Brain | 3.19E-01 | 3.19E-01 | 3.16E-01 | 3.16E-01 | 2.80E-03 | 0.071 | 0.963 | 0.918 |
| **Radial Diffusivity** |  |  |  |  |  |  |  |  |
| Caudate | 7.33E-04 | 7.38E-04 | 7.32E-04 | 7.59E-04 | 2.46E-05 | 0.423 | 0.210 | 0.397 |
| Corpus Callosum | 1.15E-03 | 1.08E-03 | 1.08E-03 | 9.86E-04 | 6.56E-05 | **0.014** | **0.021** | 0.785 |
| Cerebellum | 1.03E-03 | 1.04E-03 | 1.06E-03 | 1.03E-03 | 6.24E-05 | 0.826 | 0.729 | 0.595 |
| Left Hippocampus | 9.26E-04 | 8.96E-04 | 8.98E-04 | 8.94E-04 | 5.83E-05 | 0.610 | 0.565 | 0.663 |
| Right Hippocampus | 9.35E-04 | 9.16E-04 | 9.21E-04 | 8.92E-04 | 5.22E-05 | 0.460 | 0.364 | 0.846 |
| Internal Capsule | 6.41E-04 | 6.41E-04 | 6.44E-04 | 6.47E-04 | 1.70E-05 | 0.614 | 0.902 | 0.868 |
| Left Cortex | 8.68E-04 | 8.51E-04 | 8.56E-04 | 8.70E-04 | 3.10E-05 | 0.800 | 0.932 | 0.328 |
| Right Cortex | 8.43E-04 | 8.33E-04 | 8.58E-04 | 8.49E-04 | 2.50E-05 | 0.242 | 0.464 | 0.976 |
| Thalamus | 7.16E-04 | 7.20E-04 | 7.14E-04 | 7.15E-04 | 1.32E-05 | 0.549 | 0.707 | 0.864 |
| Total White Matter | 8.58E-04 | 8.42E-04 | 8.53E-04 | 8.60E-04 | 2.57E-05 | 0.621 | 0.739 | 0.400 |
| Whole Brain | 8.91E-04 | 8.77E-04 | 8.94E-04 | 8.97E-04 | 2.41E-05 | 0.341 | 0.649 | 0.510 |
| **Axial Diffusivity** |  |  |  |  |  |  |  |  |
| Caudate | 1.22E-03 | 1.23E-03 | 1.22E-03 | 1.22E-03 | 3.32E-05 | 0.930 | 0.746 | 0.934 |
| Corpus Callosum | 1.83E-03 | 1.71E-03 | 1.68E-03 | 1.55E-03 | 9.71E-05 | **0.004** | **0.017** | 0.966 |
| Cerebellum | 1.35E-03 | 1.35E-03 | 1.37E-03 | 1.33E-03 | 6.45E-05 | 0.942 | 0.653 | 0.540 |
| Left Hippocampus | 1.47E-03 | 1.39E-03 | 1.41E-03 | 1.37E-03 | 6.36E-05 | 0.268 | 0.069 | 0.643 |
| Right Hippocampus | 1.47E-03 | 1.43E-03 | 1.46E-03 | 1.40E-03 | 7.59E-05 | 0.565 | 0.188 | 0.839 |
| Internal Capsule | 1.28E-03 | 1.27E-03 | 1.26E-03 | 1.25E-03 | 2.06E-05 | 0.189 | 0.242 | 0.994 |
| Left Cortex | 1.40E-03 | 1.37E-03 | 1.37E-03 | 1.38E-03 | 4.23E-05 | 0.759 | 0.791 | 0.389 |
| Right Cortex | 1.37E-03 | 1.35E-03 | 1.37E-03 | 1.36E-03 | 3.17E-05 | 0.879 | 0.405 | 0.840 |
| Thalamus | 1.15E-03 | 1.16E-03 | 1.14E-03 | 1.11E-03 | 2.66E-05 | **0.019** | 0.490 | 0.171 |
| Total White Matter | 1.39E-03 | 1.36E-03 | 1.36E-03 | 1.37E-03 | 3.40E-05 | 0.693 | 0.635 | 0.407 |
| Whole Brain | 1.44E-03 | 1.42E-03 | 1.44E-03 | 1.43E-03 | 3.20E-05 | 0.751 | 0.370 | 0.535 |
| **Mean Diffusivity** |  |  |  |  |  |  |  |  |
| Caudate | 8.96E-04 | 9.00E-04 | 8.94E-04 | 9.14E-04 | 2.55E-05 | 0.631 | 0.341 | 0.560 |
| Corpus Callosum | 1.38E-03 | 1.29E-03 | 1.28E-03 | 1.18E-03 | 7.51E-05 | **0.008** | **0.018** | 0.859 |
| Cerebellum | 1.14E-03 | 1.14E-03 | 1.16E-03 | 1.13E-03 | 6.30E-05 | 0.904 | 0.702 | 0.575 |
| Left Hippocampus | 1.11E-03 | 1.06E-03 | 1.07E-03 | 1.03E-03 | 5.11E-05 | 0.217 | 0.115 | 0.902 |
| Right Hippocampus | 1.11E-03 | 1.09E-03 | 1.10E-03 | 1.06E-03 | 5.84E-05 | 0.490 | 0.266 | 0.838 |
| Internal Capsule | 8.54E-04 | 8.49E-04 | 8.46E-04 | 8.44E-04 | 1.55E-05 | 0.411 | 0.691 | 0.872 |
| Left Cortex | 1.04E-03 | 1.02E-03 | 1.03E-03 | 1.04E-03 | 3.45E-05 | 0.979 | 0.873 | 0.347 |
| Right Cortex | 1.02E-03 | 1.01E-03 | 1.03E-03 | 1.02E-03 | 2.69E-05 | 0.430 | 0.435 | 0.922 |
| Thalamus | 8.61E-04 | 8.66E-04 | 8.54E-04 | 8.52E-04 | 1.79E-05 | 0.242 | 0.857 | 0.665 |
| Total White Matter | 1.03E-03 | 1.02E-03 | 1.02E-03 | 1.03E-03 | 2.82E-05 | 0.887 | 0.694 | 0.398 |
| Whole Brain | 1.07E-03 | 1.06E-03 | 1.07E-03 | 1.07E-03 | 2.65E-05 | 0.480 | 0.524 | 0.516 |

^1^Values are means of 6-8 replicate pigs with MRI data collected at 26-29 d of age. Fractional anisotropy expressed in FA units; radial diffusivity, axial diffusivity, and mean diffusivity expressed as x 10^-3^ mm^2^ / sec.

^2^Size, main effect of birth weight (i.e. AGA vs. SGA); Diet, main effect of dietary intervention (i.e. HF vs. CON); Size*Diet, interaction effect of birth weight and dietary intervention

Abbreviations: AGA, appropriate for gestational age; SGA, small for gestational age; CON, control; HF, hydrolyzed fat. Data are presented as means and pooled SEM
