# Supplementary material for: Hydrolyzed Fat Formula Increases Brain White Matter in Small for Gestational Age and Appropriate for Gestational Age Neonatal Piglets
Source: Front Pediatr. 2020 Feb 12;8:32. doi: 10.3389/fped.2020.00032 (PMC7029735; doi:10.3389/fped.2020.00032)
Supplement: Supplementary file 3 [file Table_3.DOCX]

Supplementary Material

| **Supplementary Table 3.** Fatty acid composition of piglet hippocampus (mg/g) | | | | | | | |
| --- | --- | --- | --- | --- | --- | --- | --- |
|  | AGA | | SGA | | P-value | | |
| **FAME** | **CON** | **HF** | **CON** | **HF** | Size | Diet | Size*Diet |
| **14:0** | 0.14±.01 | 0.12±.01 | 0.14±.01 | 0.12±.01 | 0.773 | **0.004** | 0.649 |
| **16:0 DMA** | 0.68±.07 | 0.68±.09 | 0.66±.08 | 0.72±.10 | 0.758 | 0.284 | 0.251 |
| **16:0** | 4.30±.21 | 4.39±.14 | 4.33±.20 | 4.48±.22 | 0.396 | 0.082 | 0.646 |
| **16:1n9** | 0.12±.01 | 0.13±.02 | 0.13±.01 | 0.13±.01 | 0.936 | 0.726 | 0.761 |
| **16:1n7** | 0.23±.02 | 0.22±.03 | 0.24±.03 | 0.24±.04 | 0.236 | 0.907 | 0.740 |
| **18:0 DMA** | 1.05±.05 | 1.08±.08 | 1.05±.06 | 1.07±.07 | 0.614 | 0.248 | 0.785 |
| **18:1 DMA** | 0.40±.06 | 0.40±.10 | 0.39±.08 | 0.42±.09 | 0.702 | 0.604 | 0.633 |
| **18:0** | 5.65±.22 | 5.70±.28 | 5.61±.25 | 5.67±.29 | 0.728 | 0.510 | 0.955 |
| **18:1n9** | 4.05±.37 | 4.02±.50 | 3.94±.53 | 4.29±.64 | 0.664 | 0.387 | 0.296 |
| **18:1n7** | 1.20±.13 | 1.26±.13 | 1.17±.11 | 1.22±.11 | 0.401 | 0.216 | 0.855 |
| **18:2n6** | 0.32±.03 | 0.31±.03 | 0.32±.05 | 0.32±.04 | 0.624 | 0.617 | 0.483 |
| **20:0** | 0.14±.02 | 0.14±.02 | 0.14±.02 | 0.15±.03 | 0.463 | 0.219 | 0.453 |
| **20:1n9** | 0.15±.02 | 0.15±.03 | 0.15±.02 | 0.18±.05 | 0.379 | 0.236 | 0.324 |
| **20:2n6** | 0.08±.01 | 0.09±.02 | 0.08±.02 | 0.09±.02 | 0.937 | 0.188 | 0.586 |
| **20:3n6** | 0.13±.02 | 0.12±.01 | 0.12±.02 | 0.12±.01 | 0.526 | 0.094 | 0.398 |
| **20:4n6** | 2.26±.07 | 2.37±.11 | 2.25±.09 | 2.31±.14 | 0.317 | **0.028** | 0.557 |
| **22:0** | 0.21±.03 | 0.21±.05 | 0.21±.05 | 0.23±.05 | 0.713 | 0.578 | 0.555 |
| **22:1n9** | 0.08±.02 | 0.08±.03 | 0.07±.02 | 0.08±.03 | 0.990 | 0.362 | 0.498 |
| **22:2n6** | 0.09±.02 | 0.10±.03 | 0.09±.03 | 0.10±.02 | 0.792 | 0.550 | 0.843 |
| **22:4n6** | 1.07±.07 | 1.11±.04 | 1.08±.08 | 1.06±.11 | 0.529 | 0.704 | 0.254 |
| **22:5n6** | 0.48±.03 | 0.52±.09 | 0.50±.08 | 0.55±.10 | 0.382 | 0.121 | 0.864 |
| **22:5n3** | 0.05±.01 | 0.04±.01 | 0.06±.01 | 0.05±.01 | **0.008** | **<0.001** | 0.553 |
| **24:0** | 0.26±.06 | 0.27±.08 | 0.27±.09 | 0.29±.08 | 0.594 | 0.593 | 0.755 |
| **22:6n3** | 1.54±.19 | 1.50±.18 | 1.52±.19 | 1.46±.24 | 0.659 | 0.490 | 0.937 |
| **24:1n9** | 0.39±.08 | 0.41±.12 | 0.39±.10 | 0.43±.09 | 0.715 | 0.329 | 0.907 |
| **TUFA** | 0.63±.10 | 0.63±.14 | 0.59±.13 | 0.66±.15 | 0.982 | 0.409 | 0.442 |

Values presented as the means ± SEM of concentration of fatty acids of 8-9 replicate pigs collected at 26-29 d of age

^2^Size, main effect of birth weight (i.e. AGA vs. SGA); Diet, main effect of dietary intervention (i.e. HF vs. CON); Size*Diet, interaction effect of birth weight and dietary intervention.

Abbreviations: AGA, appropriate for gestational age; SGA, small for gestational age; CON, control; HF, hydrolyzed fat; FAME, fatty acid methyl ester; DMA, dimethylacetal; TUFA, total unidentified fatty acids.
