# Supplementary material for: Hydrolyzed Fat Formula Increases Brain White Matter in Small for Gestational Age and Appropriate for Gestational Age Neonatal Piglets
Source: Front Pediatr. 2020 Feb 12;8:32. doi: 10.3389/fped.2020.00032 (PMC7029735; doi:10.3389/fped.2020.00032)
Supplement: Supplementary file 4 [file Table_4.DOCX]

Supplementary Material

| **Supplementary Table 4.** Fatty acid composition of piglet cerebellum (mg/g) | | | | | | | |
| --- | --- | --- | --- | --- | --- | --- | --- |
|  | AGA | | SGA | | P-value | | |
| **FAME** | **CON** | **HF** | **CON** | **HF** | Size | Diet | Size*Diet |
| **14:0** | 0.10±.01 | 0.08±.01 | 0.09±.01 | 0.09±.10 | 0.630 | **<0.001** | 0.18 |
| **16:0 DMA** | 0.58±.07 | 0.57±.05 | 0.55±.08 | 0.57±.04 | 0.400 | 0.909 | 0.557 |
| **16:0** | 3.76±.27 | 3.81±.16 | 3.7±.27 | 3.79±.23 | 0.634 | 0.381 | 0.867 |
| **16:1n9** | 0.10±.01 | 0.11±.02 | 0.10±.01 | 0.11±.02 | 0.617 | 0.248 | 0.967 |
| **16:1n7** | 0.15±.02 | 0.16±.01 | 0.15±.03 | 0.15±.02 | 0.727 | 0.537 | 0.814 |
| **18:0 DMA** | 0.83±.08 | 0.83±.06 | 0.80±.08 | 0.80±.08 | 0.231 | 0.966 | 0.932 |
| **18:1 DMA** | 0.37±.07 | 0.35±.07 | 0.37±.08 | 0.34±.05 | 0.676 | 0.317 | 0.808 |
| **18:0** | 4.48±.39 | 4.53±.29 | 4.38±.40 | 4.41±.35 | 0.384 | 0.774 | 0.927 |
| **18:1n9** | 3.64±.46 | 3.54±.35 | 3.55±.39 | 3.45±.34 | 0.478 | 0.460 | 0.986 |
| **18:1n7** | 0.97±.15 | 0.98±.10 | 0.91±.16 | 0.93±.13 | 0.239 | 0.762 | 0.963 |
| **18:2n6** | 0.29±.04 | 0.27±.03 | 0.28±.05 | 0.27±.03 | 0.607 | 0.340 | 0.647 |
| **20:0** | 0.14±.03 | 0.13±.02 | 0.13±.03 | 0.14±.02 | 0.763 | 0.727 | 0.661 |
| **20:1n9** | 0.22±.04 | 0.21±.05 | 0.23±.06 | 0.20±.03 | 0.868 | 0.313 | 0.381 |
| **20:2n6** | 0.06±.01 | 0.06±.01 | 0.06±.01 | 0.06±.01 | 0.446 | 0.334 | 0.920 |
| **20:3n6** | 0.11±.02 | 0.10±.01 | 0.10±.02 | 0.09±.01 | 0.164 | 0.121 | 0.776 |
| **20:4n6** | 1.84±.11 | 1.94±.09 | 1.80±.19 | 1.89±.14 | 0.409 | 0.063 | 0.998 |
| **22:0** | 0.17±.05 | 0.15±.03 | 0.15±.06 | 0.15±.03 | 0.604 | 0.547 | 0.667 |
| **22:1n9** | 0.05±.02 | 0.05±.01 | 0.05±.02 | 0.05±.01 | 0.639 | 0.464 | 0.765 |
| **22:2n6** | 0.05±.01 | 0.05±.01 | 0.05±.01 | 0.05±.01 | 0.914 | 0.821 | 0.822 |
| **22:4n6** | 0.71±.07 | 0.73±.05 | 0.69±.06 | 0.72±.05 | 0.501 | 0.176 | 0.853 |
| **22:5n6** | 0.30±.04 | 0.36±.05 | 0.30±.06 | 0.38±.06 | 0.596 | **0.001** | 0.507 |
| **22:5n3** | 0.07±.01 | 0.06±.01 | 0.07±.01 | 0.06±.01 | 0.533 | **0.050** | 0.538 |
| **24:0** | 0.20±.07 | 0.18±.05 | 0.17±.07 | 0.18±.04 | 0.511 | 0.802 | 0.596 |
| **22:6n3** | 1.64±.15 | 1.62±.06 | 1.64±.15 | 1.55±.12 | 0.437 | 0.203 | 0.366 |
| **24:1n9** | 0.37±.14 | 0.36±.11 | 0.35±.14 | 0.35±.08 | 0.756 | 0.832 | 0.959 |
| **TUFA** | 0.52±.12 | 0.49±.10 | 0.49±.14 | 0.46±.08 | 0.467 | 0.429 | 0.919 |

Values presented as the means ± SEM of concentration of fatty acids of 8-9 replicate pigs collected at 26-29 d of age

^2^Size, main effect of birth weight (i.e. AGA vs. SGA); Diet, main effect of dietary intervention (i.e. HF vs. CON); Size*Diet, interaction effect of birth weight and dietary intervention.

Abbreviations: AGA, appropriate for gestational age; SGA, small for gestational age; CON, control; HF, hydrolyzed fat; FAME, fatty acid methyl ester; DMA, dimethylacetal; TUFA, total unidentified fatty acids.
