# Supplementary material for: Hydrolyzed Fat Formula Increases Brain White Matter in Small for Gestational Age and Appropriate for Gestational Age Neonatal Piglets
Source: Front Pediatr. 2020 Feb 12;8:32. doi: 10.3389/fped.2020.00032 (PMC7029735; doi:10.3389/fped.2020.00032)
Supplement: Supplementary file 5 [file Table_5.DOCX]

Supplementary Material

| **Supplementary Table 5**. Pathway analysis of up-regulated genes due to SGA in the hippocampus of 2-wk-old piglets^1^ | | | | |
| --- | --- | --- | --- | --- |
| Top Enriched Kegg Pathways | Enrichment Score | Gene Count | P-value | Benjamini value^2^ |
| Gastric acid secretion | 1.89 | 8 | 1.20E-05 | 1.80E-03 |
| Salivary secretion | 1.89 | 7 | 2.10E-04 | 1.50E-02 |
| Adrenergic signaling in cardiomyocytes | 1.89 | 9 | 3.30E-04 | 1.20E-02 |
| Insulin secretion | 1.89 | 7 | 5.20E-04 | 1.60E-02 |
| Cardiac muscle contraction | 1.89 | 6 | 1.70E-03 | 3.10E-02 |
| cAMP signaling pathway | 1.89 | 8 | 8.20E-03 | 9.10E-02 |
| Endocrine and other factor-regulated calcium reabsorption | 1.89 | 4 | 1.60E-02 | 1.40E-01 |
| Bile secretion | 1.89 | 4 | 2.30E-02 | 1.70E-01 |
| Thyroid hormone synthesis | 1.89 | 4 | 4.70E-02 | 2.60E-01 |
| Thyroid hormone signaling pathway | 1.89 | 5 | 6.20E-02 | 3.00E-01 |
| Pancreatic secretion | 1.89 | 4 | 7.10E-02 | 3.10E-01 |
| Aldosterone-regulated sodium reabsorption | 1.89 | 3 | 9.60E-02 | 3.70E-01 |
| Gastric acid secretion | 1.64 | 8 | 1.20E-05 | 1.80E-03 |
| Aldosterone synthesis and secretion | 1.64 | 7 | 3.20E-04 | 1.60E-02 |
| Adrenergic signaling in cardiomyocytes | 1.64 | 9 | 3.30E-04 | 1.20E-02 |
| Insulin secretion | 1.64 | 7 | 5.20E-04 | 1.60E-02 |
| Calcium signaling pathway | 1.64 | 9 | 1.10E-03 | 2.70E-02 |
| Circadian entrainment | 1.64 | 7 | 1.40E-03 | 2.90E-02 |
| Olfactory transduction | 1.64 | 4 | 2.40E-03 | 4.00E-02 |
| Oxytocin signaling pathway | 1.64 | 8 | 3.70E-03 | 5.40E-02 |
| Melanogenesis | 1.64 | 6 | 5.30E-03 | 7.00E-02 |
| Amphetamine addiction | 1.64 | 5 | 8.10E-03 | 9.60E-02 |
| cAMP signaling pathway | 1.64 | 8 | 8.20E-03 | 9.10E-02 |
| Long-term potentiation | 1.64 | 5 | 8.60E-03 | 8.80E-02 |
| Cholinergic synapse | 1.64 | 6 | 1.30E-02 | 1.20E-01 |
| Dopaminergic synapse | 1.64 | 6 | 1.70E-02 | 1.40E-01 |
| Glucagon signaling pathway | 1.64 | 5 | 2.00E-02 | 1.60E-01 |
| Inflammatory mediator regulation of TRP channels | 1.64 | 5 | 2.90E-02 | 2.00E-01 |
| Retrograde endocannabinoid signaling | 1.64 | 5 | 3.30E-02 | 2.10E-01 |
| Glioma | 1.64 | 4 | 4.10E-02 | 2.40E-01 |
| Glutamatergic synapse | 1.64 | 5 | 6.40E-02 | 3.00E-01 |
| Wnt signaling pathway | 1.64 | 5 | 7.00E-02 | 3.10E-01 |
| GnRH signaling pathway | 1.64 | 4 | 8.70E-02 | 3.60E-01 |
| Proteoglycans in cancer | 1.64 | 6 | 9.30E-02 | 3.70E-01 |
| Tuberculosis | 1.64 | 5 | 1.10E-01 | 3.80E-01 |
| Oocyte meiosis | 1.64 | 4 | 1.50E-01 | 4.80E-01 |
| Vascular smooth muscle contraction | 1.64 | 4 | 1.60E-01 | 5.00E-01 |
| Neurotrophin signaling pathway | 1.64 | 4 | 1.90E-01 | 5.60E-01 |
| GABAergic synapse | 1.64 | 3 | 2.70E-01 | 6.70E-01 |
| ErbB signaling pathway | 1.64 | 3 | 3.00E-01 | 6.90E-01 |
| Morphine addiction | 1.64 | 3 | 3.00E-01 | 6.90E-01 |
| Estrogen signaling pathway | 1.64 | 3 | 3.50E-01 | 7.40E-01 |
| HIF-1 signaling pathway | 1.64 | 3 | 3.50E-01 | 7.40E-01 |
| Serotonergic synapse | 1.64 | 3 | 3.70E-01 | 7.50E-01 |
| Rap1 signaling pathway | 1.64 | 4 | 4.60E-01 | 8.20E-01 |
| Ras signaling pathway | 1.64 | 4 | 4.90E-01 | 8.40E-01 |

^1^Enrichment scores as determined by Functional Annotation Clustering in DAVID v.6.8, which rank overall importance of the annotation term groups.

^2^Main effect of birth weight (i.e. SGA vs. AGA); the Benjamini value corrects for multiple comparisons

Abbreviations: AGA, appropriate for gestational age; SGA, small for gestational age
