# Supplementary material for: Hydrolyzed Fat Formula Increases Brain White Matter in Small for Gestational Age and Appropriate for Gestational Age Neonatal Piglets
Source: Front Pediatr. 2020 Feb 12;8:32. doi: 10.3389/fped.2020.00032 (PMC7029735; doi:10.3389/fped.2020.00032)
Supplement: Supplementary file 6 [file Data_Sheet_1.docx]

Supplementary Material


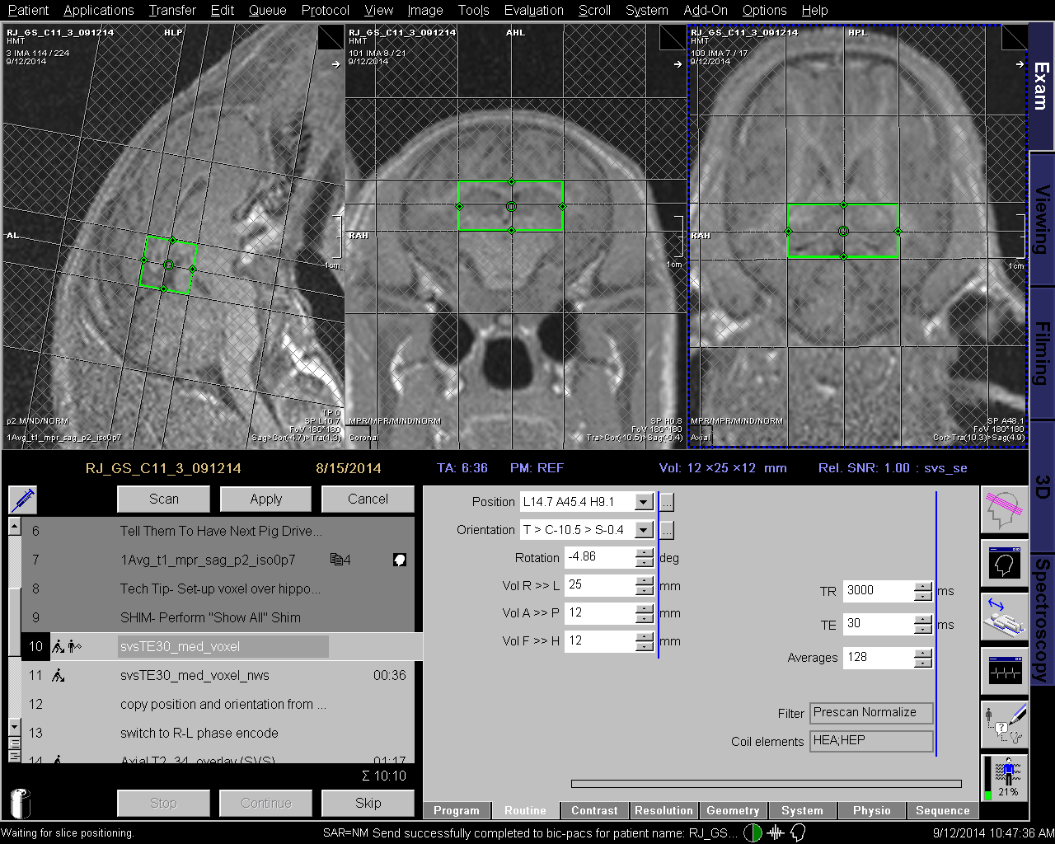


**Supplementary Figure 1**. Representative placement of the single voxel spectroscopy over the hippocampi. The cross-hatched regions show regional saturation bands.
