# Supplementary material for: Hydrolyzed Fat Formula Increases Brain White Matter in Small for Gestational Age and Appropriate for Gestational Age Neonatal Piglets
Source: Front Pediatr. 2020 Feb 12;8:32. doi: 10.3389/fped.2020.00032 (PMC7029735; doi:10.3389/fped.2020.00032)
Supplement: Supplementary file 7 [file Data_Sheet_2.docx]

Supplementary Material


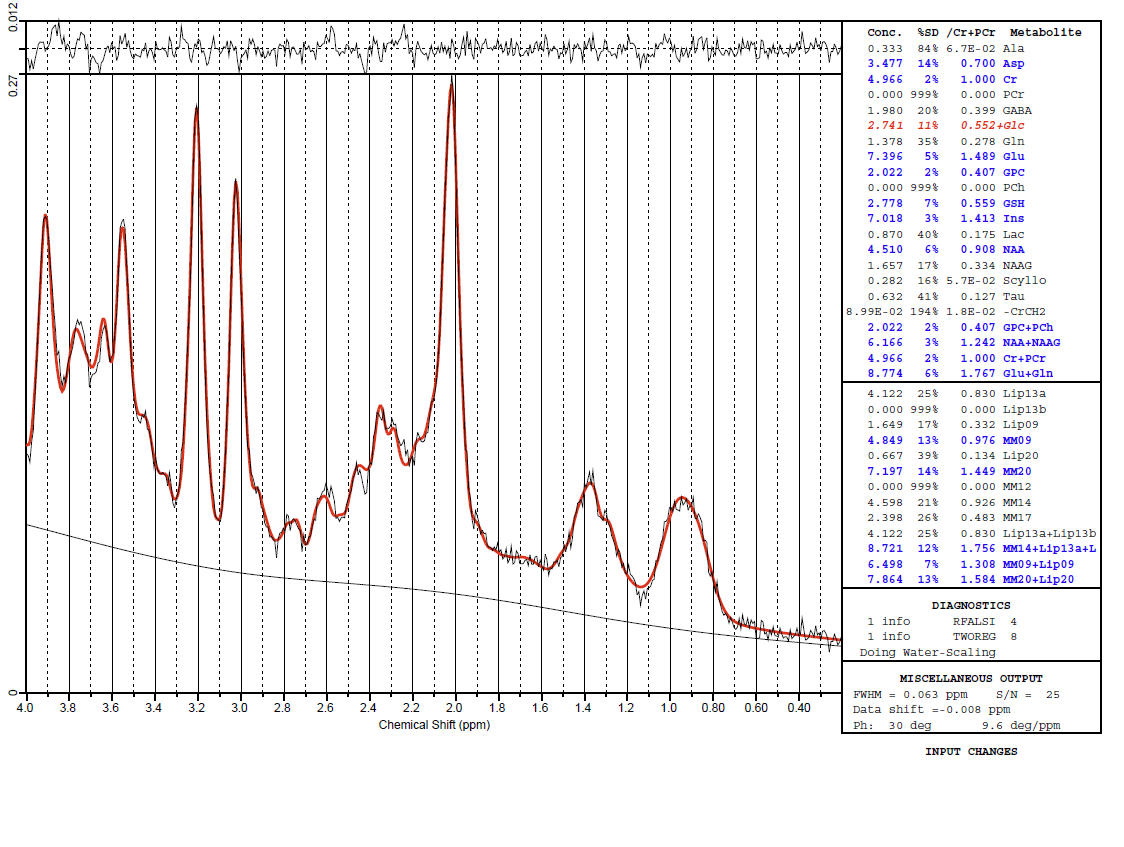


**Supplementary Figure 2**. Representative example of piglet magnetic resonance spectroscopy spectra.
